# The Loss of YTHDC1 in Gut Macrophages Exacerbates Inflammatory Bowel Disease

**DOI:** 10.1002/advs.202205620

**Published:** 2023-03-15

**Authors:** Xuejun Ge, Gang Xue, Yan Ding, Ran Li, Kaining Hu, Tengjiao Xu, Ming Sun, Wang Liao, Bin Zhao, Chuangyu Wen, Jie Du

**Affiliations:** ^1^ Shanxi Province Key Laboratory of Oral Diseases Prevention and New Materials Shanxi Medical University School and Hospital of Stomatology Taiyuan Shanxi 030001 China; ^2^ Department of Gastroenterology Second Hospital of Shanxi Medical University Taiyuan Shanxi 030001 China; ^3^ Department of Dermatology Hainan Provincial Hospital of Skin Disease Haikou Hainan 570000 China; ^4^ Department of Dermatology Hainan Medical University Affiliated Dermatology Hospital of Hainan Medical College Haikou Hainan 570000 China; ^5^ Department of Human Genetics The University of Chicago Chicago IL 60637 USA; ^6^ College of Life Sciences Mudanjiang Medical University Mudanjiang Heilongjiang 157011 China; ^7^ Department of Cardiology Hainan General Hospital and Hainan Affiliated Hospital of Hainan Medical University Haikou 570311 China; ^8^ Central Laboratory Affiliated Dongguan Hospital Southern Medical University Dongguan Guangdong 523108 China; ^9^ Institute of Biomedical Research Shanxi Medical University Taiyuan Shanxi 030001 China

**Keywords:** inflammatory bowel disease, macrophage, Nme1, Rhoh, YTHDC1

## Abstract

The nuclear *N* ^6^‐methyladenosine (m^6^A) reader YT521‐B homology‐domain‐containing protein 1 (YTHDC1) is required to maintain embryonic stem cell identity. However, little is known about its biological functions in intestinal‐resident macrophages and inflammatory bowel disease (IBD). Herein, it is demonstrated that macrophage‐specific depletion or insufficiency of YTHDC1 accelerates IBD development in animal models. On the molecular basis, YTHDC1 reduction in IBD‐derived macrophages is attributed to Zinc finger protein 36 (ZFP36)‐induced mRNA degradation. Importantly, transcriptome profiling and mechanistic assays unveil that YTHDC1 in macrophages regulates Ras homolog family member H (RHOH) to suppress inflammatory responses and fine‐tunes NME nucleoside diphosphate kinase 1 (NME1) to enhance the integrity of colonic epithelial barrier, respectively. Collectively, this study identifies YTHDC1 as an important factor for the resolution of inflammatory responses and restoration of colonic epithelial barrier in the setting of IBD.

## Graphical abstract

Inflammatory bowel disease is identified by chronic and relapsing inflammation in the intestinal tract. It is revealed that YT521‐B homology‐domain‐containing protein 1 (YTHDC1) in gut macrophages is a novel factor responsible for the development of inflammatory bowel disease. Targeting YTHDC1 in gut macrophages might be an effective method for inflammatory bowel disease management.

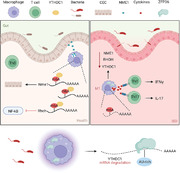

## 1 Introduction

YT521‐B homology‐domain‐containing protein 1 (YTHDC1), a nuclear‐localized factor, is well known for its functions in regulating *N* ^6^‐methyladenosine (m^6^A)‐modified RNAs.^[^ ^1^ ^]^ As a nuclear m^6^A reader, YTHDC1 is identified to fine‐tune nuclear mRNA processing events such as transcription, alternative splicing, degradation, and nuclear export.^[^ ^1^, ^2^ ^]^ The nuclear noncoding RNAs including metastasis associated lung adenocarcinoma transcript 1 (MALAT1) and X inactive specific transcript (XIST) are the major target of YTHDC1 confirmed by cross‐linking and immunoprecipitation (CLIP) studies.^[^ ^3^ ^]^ The preferential enrichment of YTHDC1 on nuclear ncRNAs indicates the long time of ncRNAs residence in the nucleus in contrast to the short time of mRNAs.^[^ ^3^, ^4^ ^]^ However, YTHDC1 can still interact with mRNAs amid or post transcription, which thus fosters YTHDC1 to modulate nuclear mRNA processing.^[^ ^4^ ^]^ In addition, recent studies have reported YTHDC1 mediates chromosome‐associated RNAs and influences chromatin state and transcription in mouse embryonic stem (ES) cells.^[^ ^5^ ^]^ Elimination of YTHDC1 impedes the maintenance of mouse ES cell identity and blocks the self‐renewal of leukemia stem cells (LSCs),^[^ ^6^ ^]^ highlighting its multifaceted effects on cell fate. To date, the functions of YTHDC1 in immune cells as well as inflammatory bowel disease (IBD) are poorly understood.

IBD that encompasses ulcerative colitis (UC) and Crohn disease (CD) is identified by chronic and relapsing inflammation in the intestinal tract, leading to immune‐regulated tissue damage and intestinal barrier dysfunction.^[^ ^7^ ^]^ UC mainly involves the colonic mucosa with confluent inflammation, while CD can penetrate transmurally and occur anywhere in the intestinal tract.^[^ ^8^ ^]^ Albeit the precise pathogenesis of IBD remains unclear, people accept that IBD develops via a convergence of immunological, microbial, environmental, and genetically susceptible factors. So far, due to the shortage of known cure for IBD, current management is focused on symptom control and achievement of long‐term remission.^[^ ^7^ ^]^ A critical physiological process maintaining intestinal homeostasis is the controlling of inflammatory response.^[^ ^9^ ^]^ Both innate immunity and excessive adaptive immune responses are identified to trigger colonic inflammation.^[^ ^9a^ ^]^ Importantly, CD4^+^ T helper (Th)1 and Th17 cells are thought to be the key factors responsible for driving mucosal inflammation.^[^ ^9a^ ^]^ Intestinal epithelial cells (IECs) play an important role in the resolution of inflammation because of their anatomical location as well as complicated communication(s) with the immune system of lamina propria and the local gut microbiota.^[^ ^9^, ^10^ ^]^

Macrophages that reside in intestinal tissues have key functions in mediating the integrity of the intestinal epithelial barrier and maintaining the mucosal immune homeostasis. Macrophages can primarily polarize into two subtypes based on the microenvironmental cues: the classically activated (M1) macrophages or alternatively activated (M2) ones.^[^ ^11^ ^]^ M1 macrophages are able to secret a variety of proinflammatory cytokines such as interleukin‐6 (IL‐6) and tumor necrosis factor‐alpha (TNF‐*α*) in response to interferon‐gamma (IFN‐*γ*) or lipopolysaccharide (LPS), leading to the exacerbation of IBD development. On the contrary, M2 macrophages are capable of producing anti‐inflammatory cytokines such as interleukin‐10 (IL‐10) upon interleukin‐4 (IL‐4) and interleukin‐13 (IL‐13) challenges, promoting tissue regeneration and relieving inflammation of IBD.^[^ ^11^ ^]^ Colonic tissues derived from IBD patients and animal colitis models are reported to be infiltrated with massive macrophages compared to healthy samples.^[^ ^11^, ^12^ ^]^ Due to the imbalance of M1/M2 ratio of macrophages in colonic mucosa amid the development of IBD, macrophage polarization has been considered to be a therapeutic target candidate for IBD.^[^ ^13^ ^]^ To this end, exploring the molecular mechanism underlying colonic tissue‐resident macrophage polarization during the development of IBD is urgent.

In this study, we demonstrate a comprehensive report explaining the status and potential roles of YTHDC1 of gut macrophages in the context of IBD. Our findings show the loss of YTHDC1 in the tissue‐resident macrophages aggravates IBD severity.

## 2 Results

### 2.1 The Lack of YTHDC1 has No Effects on Macrophage Development and Homeostasis of Colon Tissues

Although many studies have indicated that YTHDC1 exerts a wide range of regulatory functions in stem cells,^[^ ^5^, ^6^ ^]^ its importance in the differentiation and biological processes of macrophages remains elusive. Here, we crossed *Ythdc1* ^flox/flox^ mice with LysM‐Cre mice to generate *Ythdc1* ^flox/flox^;Lysm‐Cre mice (hereafter referred to as m*Ythdc1* ^−/−^) with conditional *Ythdc1* gene knockout in myeloid cells, the deletion of YTHDC1 protein was confirmed in both bone marrow‐derived macrophages (BMDMs) and peritoneal macrophages (PMs) harvested from m*Ythdc1* ^−/−^ mice (Figure S1a, Supporting Information). Of note, the depletion of YTHDC1 did not affect bone marrow differentiation into macrophages and cell viability of PMs (Figure S1b,c, Supporting Information). Due to primary macrophage's inability to proliferate, we applied CRISPR‐cas9 system to ablate *Ythdc1* gene in Raw264.7 cells and validated that YTHDC1 ablation has no effect on cell proliferation (Figure S1d,e, Supporting Information). Since tissue‐resident macrophages could influence immune system homeostasis and self‐renewal of epithelial cells in the colon,^[^ ^14^ ^]^ we questioned whether the deletion of YTHDC1 in macrophages has a positive or negative effect on colon tissues. The expression of LysM‐Cre enzyme was confirmed by tdTomato fluorescence and ablation of YTHDC1 was validated by immunofluorescence staining and western blot, respectively (Figure S1f,g, Supporting Information). As shown, the histological examination exhibited no differences between the wild‐type and conditional knockout mice (Figure S1h, Supporting Information). The immune cell composition in gut lamina propria and gut‐draining lymph node, cytokines, and IgA status in colonic mucosae and levels of fluorescein isothiocyanate (FITC)‐dextran in serums are comparable upon *Ythdc1* deletion (Figure S1i–m, Supporting Information).

### 2.2 The Knockout of *Ythdc1* in Macrophages Influences Colitis Development in the Inflammatory Bowel Disease Animal Models

**Figure 1:**
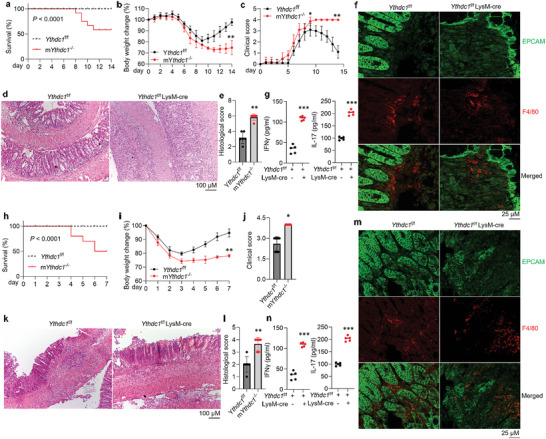
The lack of YTHDC1 in macrophages influences colitis development. a–c) Survival rate (a), body weight change (b), and clinical score (c) of *Ythdc1* ^f/f^ and m*Ythdc1* ^−/−^ mice in the DSS‐induced colitis model. *n* = 10 for each group. d–e) HE staining (d) showing the epithelial cell damage and mucosal ulceration, and histological score (e) assessing colitis severity in *Ythdc1* ^f/f^ and m*Ythdc1* ^−/−^ mice on day 9 post‐DSS administration. *n* = 6 for each group. f–g) Accumulation of F4/80^+^macrophages tested by immunostaining (f), and IFN‐*γ* and IL‐17 levels tested by ELISA (g) in the mucosae of *Ythdc1* ^f/f^ and m*Ythdc1* ^−/−^ mice on day 9 post‐DSS administration. *n* = 5 for each group. h–j) Survival rate (h), body weight change (i), and clinical score (j, on day 3) of *Ythdc1* ^f/f^ and m*Ythdc1* ^−/−^ mice in the TNBS‐induced colitis model. *n* = 10 for each group. k,l) HE staining (k) showing the epithelial cell damage and mucosal ulceration, and histological score (l) assessing colitis severity in *Ythdc1* ^f/f^ and m*Ythdc1* ^−/−^ mice on day 3 post‐TNBS administration. *n* = 6 for each group. m,n) Infiltration of F4/80^+^macrophages tested by immunostaining (m), and IFN‐*γ* and IL‐17 levels tested by ELISA (n) in the mucosae of *Ythdc1* ^f/f^ and m*Ythdc1* ^−/−^ mice on day 3 post‐TNBS administration. *n* = 5 for each group.*p < 0.05, ^**^ *p* < 0.01, ^***^ *p* < 0.001 versus corresponding control group. Data depict mean ± SD. The log‐rank test, two‐tailed Student's *t*‐test, and two‐way ANOVA were performed for statistical analyses.

To reveal the functions of macrophages with YTHDC1 depletion in the setting of IBD, both *Ythdc1* ^f/f^ and m*Ythdc1* ^−/−^ mice were treated with 2.5% dextran sulfate sodium (DSS) to induce an acute colitis model. Interestingly, the death rate, body weight loss, and clinical score were increased in m*Ythdc1* ^−/−^ mice upon DSS treatment in comparison to *Ythdc1* ^f/f^ controls (**Figure** **1**a–c). The loss of epithelial cells, lymphocyte infiltration, and damage of colonic epithelial barrier integrity were more severe in DSS‐treated m*Ythdc1* ^−/−^ mice (Figure 1d and Figure S1n, Supporting Information), accompanied by higher histological score (Figure 1e). Massive macrophages were present in the lamina propria of mice developing colitis (Figure 1f and Figure S1p, Supporting Information). Next, we detected the influence of macrophage YTHDC1 expression in Th cell differentiation in both gut lamina propria and lymph node by fluorescence‐activated cell sorting (FACS) analysis (Figure S1r,s, Supporting Information). As shown, increased proportions of Th1 and Th17 T cells and decreased proportions of Treg and IL‐10^+^ T cells were observed in m*Ythdc1* ^−/−^ mice under colitis conditions (Figure S2a, Supporting Information), so did cytokines levels in colonic mucosae (Figure 1g and Figure S2b, Supporting Information). Next, we used 2,4,6‐trinitrobenzene sulfonic acid (TNBS) to establish another chemically induced colitis and observed increased death rate, body weight loss, clinical and histological scores, and inflammatory responses in m*Ythdc1* ^−/−^ mice accordantly (Figure 1h–n and Figures S1o,q and S2c,d, Supporting Information).

*Il10*‐knockout (*Il10^−/−^*) mice develop spontaneous colitis by 2–4 months of age.^[^ ^15^ ^]^ To further validate this phenotype, we crossed *Ythdc1* ^f/f^ or m*Ythdc1* ^−/−^ mice with *Il10* ^−/−^ mice to detect the roles of YTHDC1 in a spontaneous colitis model. The deletion of IL10 protein was verified by western blot (Figure S2e, Supporting Information). Consistently, upon *Ythdc1* deletion in macrophages, ≈50% of *Il10* ^−/−^ mice died within 14 weeks compared to the 100% survival rate of the control group (Figure S2f, Supporting Information). In addition, more lymphocyte infiltration, higher Th1 and Th17 and lower Treg and IL‐10^+^ T cells frequencies, and twofold increases of IFN‐*γ* and interleukin‐17 (IL‐17) secretions were observed in m*Ythdc1* ^−/−^ mice with *Il10* ^−/−^ background (Figure S2g–i, Supporting Information). To confirm that the severe colitis of m*Ythdc1* ^−/−^ mice resulted from defects in hematopoietic cells, bone marrow (BM) transplantation (BMT) was performed, and the recipient mice were analyzed 8 weeks post‐transplantation. As shown, either in the parallel BMT experiment (*Ythdc1* ^f/f^ BM to *Ythdc1* ^f/f^ mice; m*Ythdc1* ^−/−^ BM to m*Ythdc1* ^−/−^ mice) or in the cross BMT experiment (*Ythdc1* ^f/f^ BM to m*Ythdc1* ^−/−^ mice; m*Ythdc1* ^−/−^ BM to *Ythdc1* ^f/f^ mice), the colitis progression of the recipient mice was dominated by bone marrow from the donor mice, but not by the original genotype of recipient mice (Figure S2j–n, Supporting Information), revealing the colitis development is of hematopoietic origin. Since LysM‐Cre is also reported to be expressed in neutrophils,^[^ ^16^ ^]^ we crossed *Ythdc1* ^f/f^ mice with S100a8‐Cre transgenic mice to ablate *Ythdc1* in neutrophils specifically. Our data illustrated that conditional knockout of *Ythdc1* in neutrophils did not affect the development of colitis (Figure S2o–r, Supporting Information), confirming that the colitis phenotype of m*Ythdc1* ^−/−^ mice was not attributed to neutrophils. To further explain the mechanism by which macrophage *Ythdc1* deletion affects Th cell differentiation, we detected the expression of interleukin‐12 (*Il‐12)* and interleukin‐13 (*Il‐23*) that are responsible for the differentiation of T cells. Importantly, we observed higher levels of *Il‐12* and *Il‐23* in gut macrophages from m*Ythdc1* ^−/−^ mice under colitis conditions (Figure S2s, Supporting Information).

### 2.3 YTHDC1 Expression is Decreased in the Macrophages from Colonic Mucosae of IBD

**Figure 2:**
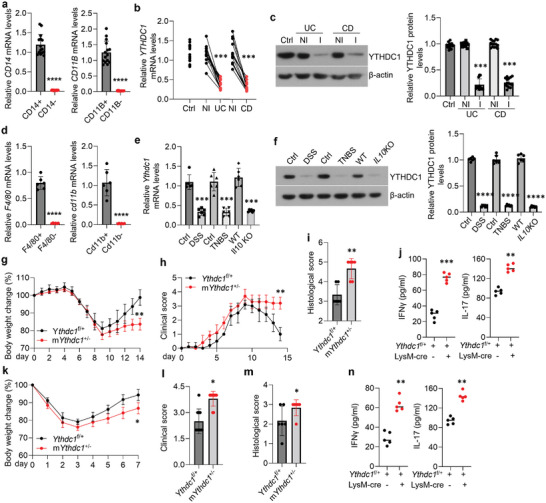
YTHDC1 in gut macrophages is decreased in the context of IBD. a) Human gut‐derived macrophages were purified by CD14 and CD11B magnetic beads. *CD14* and *CD11B* mRNA levels in the CD14^+^/CD14^−^ cells and CD11B^+^/CD11B^−^ cells were tested by qPCR. *n* = 15 for each group. b,c) Levels of YTHDC1 in purified CD14^+^CD11B^+^macrophages from healthy individuals and IBD patients were tested by qPCR (b) and western blot (c). *n* = 15 for each group. d) Mouse intestinal‐derived macrophages were purified by F4/80 and Cd11b magnetic beads. *F4/80* and *Cd11b* mRNA levels in the F4/80^+^/F4/80^−^ cells and Cd11b^+^/Cd11b^−^ cells were tested by qPCR. *n* = 6 for each group. e,f) Levels of YTHDC1 in purified F4/80^+^Cd11b^+^macrophages from control and colitis mice were tested by qPCR (e) and western blot (f). *n* = 6 for each group. g,h) Body weight change (g) and clinical score (h) of *Ythdc1* ^f/+^ and m*Ythdc1* ^+/−^ mice in the DSS‐induced colitis model. *n* = 10 for each group. i) Histological score of *Ythdc1* ^f/+^ and m*Ythdc1* ^+/−^ mice on day 9 post DSS administration. *n* = 6 for each group. j) IFN‐*γ* and IL‐17 levels tested by ELISA in the mucosae of *Ythdc1* ^f/+^ and m*Ythdc1* ^+/−^ mice on day 9 post‐DSS administration. *n* = 5 for each group. k,l) Body weight change (k) and clinical score (l, on day 3) of *Ythdc1* ^f/+^ and m*Ythdc1* ^+/−^ mice in the TNBS‐induced colitis model. *n* = 10 for each group. m) Histological score of *Ythdc1* ^f/+^ and m*Ythdc1* ^+/−^ mice on day 3 post‐TNBS administration. *n* = 6 for each group. n) IFN‐*γ* and IL‐17 levels tested by ELISA in the mucosae of *Ythdc1* ^f/+^ and m*Ythdc1* ^+/−^ mice on day 3 post‐TNBS administration. *n* = 5 for each group. Ctrl, control; WT, wild‐type; NI, non‐inflammation; I, inflammation; UC, ulcerative colitis; CD, Crohn disease; TNBS, 2,4,6‐trinitrobenzene sulfonic acid; DSS, dextran sulfate sodium; KO, knockout. *p < 0.05, ^**^ *p* < 0.01, ^***^ *p* < 0.001, ^****^ *p* < 0.0001 versus corresponding control group. Data depict mean ± SD. Two‐tailed Student's *t*‐test, one‐way ANOVA, and two‐way ANOVA were performed for statistical analyses.

Owing to the important protective roles of YTHDC1 in colitis development, we next sought to determine the levels of YTHDC1 in the macrophages derived from colonic tissues of IBD. We collected healthy colonic samples from individuals without IBD, and both inflamed and adjacent non‐inflamed colon specimens from patients being affected by IBD (Figure S3a, Supporting Information). CD14^+^CD11B^+^ macrophages in these human samples were purified by microbeads and confirmed using qPCR (**Figure** **2**a). As manifested, YTHDC1 showed a significant decrease in these enriched macrophages from IBD patients (Figure 2b,c), accompanied by reverse correlations with cytokines status in the diseased samples (Figure S3b,c, Supporting Information). Accordingly, YTHDC1 levels were also down‐regulated in the purified F4/80^+^Cd11b^+^macrophages from colitis animals compared to those of healthy controls (Figure 2d–f). These results were further confirmed in colonic macrophages enriched by the FACS technology (Figure S3d–g, Supporting Information). To resemble the insufficient, but not deleted, status of YTHDC1 in colitis, we crossed *Ythdc1* ^f/+^ mice with LysM‐Cre mice to generate *Ythdc1* ^flox/+^;Lysm‐Cre mice (hereafter referred to as m*Ythdc1* ^+/−^) with deficient YTHDC1 in macrophages (Figure S3h, Supporting Information). Notably, macrophages with insufficient YTHDC1 accelerated colitis progression in both DSS‐induced (Figure 2g–j and Figure S3i,j, Supporting Information) and TNBS‐triggered (Figure 2k–n and Figure S3k,l, Supporting Information) animal models. Together, these results exhibited that sufficient YTHDC1 in macrophages is required to protect from colitis.

### 2.4 Zinc Finger Protein 36 (ZFP36) Contributes to *Ythdc1* mRNA Degradation in Activated Macrophages

**Figure 3:**
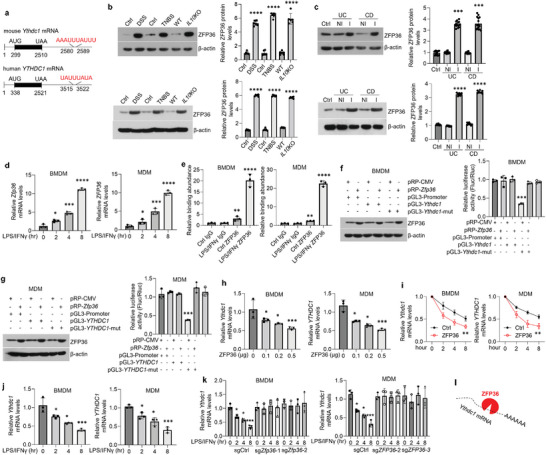
ZFP36 promotes *Ythdc1* mRNA degradation. a) Schematic illustration of AU‐rich binding site in mouse *Ythdc1* or human *YTHDC1* mRNA. b,c) Western blot and quantitative analyses showing levels of ZFP36 in magnetic‐activated cell sorting (MACS)‐enriched gut macrophages (up) or FACS‐enriched gut macrophages (bottom) from mice (b) or human (c). *n* = 6 for mice groups and *n* = 15 for human groups. d) Quantitative PCR showing *Zfp36*/*ZFP36* mRNA levels in BMDMs or MDMs upon 100 ng mL^−1^ LPS/IFN‐*γ* treatments at different time points as indicated. *n* = 3 for each group. e) CLIP assays against IgG or ZFP36 antibodies showing ZFP36 binding to the AU‐rich site in the BMDMs (left) or MDMs (right) with or without 8‐h 100 ng mL^−1^ LPS/IFN‐*γ* treatment. *n* = 3 for each group. f,g) Western blot detection of ZFP36 (left) and luciferase activity assays (right) in BMDMs (f) or MDMs (g) with various co‐transfected plasmids as indicated. h) Quantitative PCR showing *Ythdc1*/*YTHDC1* mRNA levels in BMDMs (left) or MDMs (right) with various doses of ZFP36 plasmids transfection. Plasmids were transfected using Lipofectamin 3000 for 36 h. *n* = 3 for each group. i) *Ythdc1*/*YTHDC1* mRNA decay in BMDMs (left) or MDMs (right) with 36‐h ZFP36 plasmids transfection (0.5 µg). *n* = 3 for each group. j) Quantitative PCR showing *Ythdc1*/*YTHDC1* mRNA levels in BMDMs (left) or MDMs (right) upon 100 ng mL^−1^ LPS/IFN‐*γ* treatments at different time points as indicated. *n* = 3 for each group. k) Quantitative PCR showing *Ythdc1*/*YTHDC1* mRNA levels in sgCtrl‐ or sg*Zfp36*/*ZFP36*‐transfected BMDMs (left) or MDMs (right) upon 100 ng mL^−1^ LPS/IFN‐*γ* treatments at different time points as indicated. *n* = 3 for each group. Ctrl, control; WT, wild‐type; NI, non‐inflammation; I, inflammation; UC, ulcerative colitis; CD, Crohn disease; TNBS, 2,4,6‐trinitrobenzene sulfonic acid; DSS, dextran sulfate sodium; BMDM, bone marrow‐derived macrophage; MDM, monocyte‐derived macrophage. *p < 0.05, ^**^ *p* < 0.01, ^***^ *p* < 0.001, ^****^ *p* < 0.0001 versus corresponding control group. Data depict mean ± SD. Two‐tailed Student's *t*‐test, one‐way ANOVA, and two‐way ANOVA were performed for statistical analyses.

*Ythdc1* mRNA showed a robust decrease in the context of colitis (Figure 2e). Methyltransferase like‐14 (METTL14) is a critical regulatory factor for m^6^A‐modified RNAs.^[^ ^4^ ^]^ To further figure out the mechanisms underlying YTHDC1 reduction in macrophages, we first detected *Ythdc1* mRNA levels in macrophages with *Mettl14* depletion to investigate whether *Ythdc1* mRNA is regulated by m^6^A. Our data showed that mRNA levels of *Ythdc1* were not influenced by RNA methylation (Figure S4a, Supporting Information). RNA degradation is mainly mediated via the AU‐rich element (ARE, AUUUA) which interacts with RNA‐binding proteins.^[^ ^17^ ^]^ Interestingly, we identified a conserved ARE in the 3’UTR of both mouse *Ythdc1* and human *YTHDC1* mRNAs (**Figure** **3**a). Among the ARE‐binding proteins responsible for destabilizing mRNAs,^[^ ^17^ ^]^ only ZFP36 is considerably increased in the macrophages purified from human or mouse diseased samples (Figure S4b,c, Supporting Information, and Figure 3b,c). ZFP36 is highly elevated in macrophages exposed to LPS/IFN‐*γ* (Figure 3d), as described by other studies.^[^ ^18^ ^]^ We next validated that LPS/IFN*γ* treatment indeed robustly enhanced ZFP36 binding to the ARE using CLIP‐qPCR assays (Figure 3e). In the luciferase assays, we confirmed that ZFP36 overexpression suppressed luciferase activity of macrophages transfected with pGL3‐Ythdc1/YTHDC1 plasmids but not the mutations (Figure 3f,g). Moreover, forced expression of ZFP36 decreased *Ythdc1*/*YTHDC1* mRNAs in a dose‐dependent manner (Figure 3h), and promoted *Ythdc1*/*YTHDC1* mRNAs decay (Figure 3i). Similarly, addition of LPS/IFN‐*γ* reduced *Ythdc1*/*YTHDC1* mRNAs levels in a time‐dependent fashion (Figure 3j). To survey whether the decreases of *Ythdc1*/*YTHDC1* mRNAs caused by LPS/IFN‐*γ* relies on ZFP36 signaling, we ablated ZFP36 in both human and mouse macrophages using the CRISPR‐cas9 tool (Figure S4d, Supporting Information). As shown, LPS/IFN‐*γ* have an inability to reduce *Ythdc1*/*YTHDC1* mRNAs upon ZFP36 deletion (Figure 3k). For a better understanding of ZFP36's roles in vivo, we crossed *Zfp36* ^f/f^ mice with Lysm‐Cre mice to generate *Zfp36* ^f/f^;Lysm‐Cre mice (hereafter referred to as m*Zfp36* ^−/−^). The depletion of ZFP36 in BMDMs was confirmed by western blot (Figure S4e, Supporting Information). Consistently, conditional knockout of *Zfp36* gene in macrophages ameliorated colitis development in a DSS‐induced animal model (Figure S4f–j, Supporting Information). Meanwhile, gut macrophage *Ythdc1* levels were not influenced in mice with DSS treatment upon *Zfp36* depletion (Figure S4k, Supporting Information) and there was a negative correlation between ZFP36 and YTHDC1 expression in the macrophages derived from UC or CD samples (Figure S4l,m, Supporting Information). In summary, ZFP36 is the contributing factor accounting for *Ythdc1* mRNA degradation (Figure 3l).

### 2.5 YTHDC1 Protects Mice from Colitis in an m^6^A‐Dependent Way

YTHDC1 is well known as a reader recognizing m^6^A modification on RNAs via the YTH domain.^[^ ^1b^ ^]^ To determine whether YTHDC1 in macrophages prevents colitis dependent on m^6^A RNA methylation, we constructed two identified YTH m^6^A‐binding‐site mutants^[^ ^6a^ ^]^ of YTHDC1 (W378A and W429A) and transduced them into *Ythdc1*‐depleted BMDMs with validation (Figure S5a). Next, we eliminated endogenous macrophages in wild‐type mice using clodronate and reconstituted by various BMDMs with or without lentivirus infection^[^ ^19^ ^]^ followed by TNBS administration (Figure S5b, Supporting Information). Following clodronate treatment, the entire experiment should be completed in 7 days because the mouse would be repopulated with endogenous macrophages within 1–2 weeks.^[^ ^19^ ^]^ Given that, TNBS‐induced acute colitis model is suitable for this macrophage depletion and reconstitution system instead of others. As displayed, mice reconstituted with BMDMs overexpressing wild‐type YTHDC1 dramatically attenuated colitis development and cytokines production after TNBS challenge, whereas those injected with BMDMs overexpressing mutated YTHDC1 failed to protect mice from colitis development (Figure S5c–g, Supporting Information), suggesting YTHDC1 plays a protective role in colitis dependent on m^6^A. These results were further confirmed in a DSS‐induced colitis model using *Mettl14* ^f/f^ and *Mettl14* ^f/f^;Lysm‐Cre mice (Figure S5h‐m) or using methyltransferase like‐3 (*Mettl3)* ^f/f^ and *Mettl3* ^f/f^;Lysm‐Cre mice (Figure S5n–s, Supporting Information).

### 2.6 *Ras Homolog Family Member H (Rhoh)* and NME Nucleoside Diphosphate Kinase 1 (*Nme1)* are the Downstream Targets of YTHDC1

**Figure 4:**
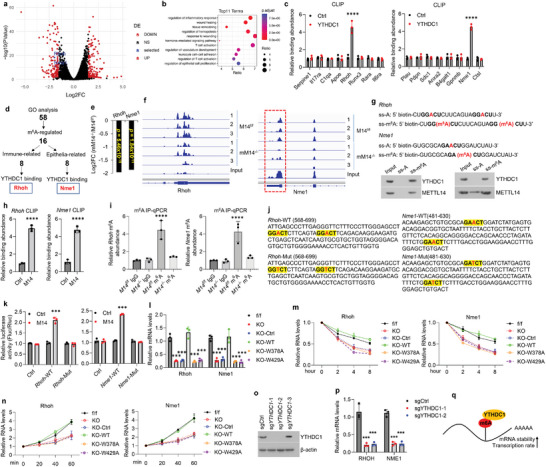
YTHDC1 regulates *Rhoh* and *Nme1* expression in macrophages. a) Volcano plot showing the differential gene expression (M14^−/−^/M14^f/f^) of M14^f/f^ and M14^−/−^ BMDMs from the online RNA‐sequencing data. b) Top 11 pathways identified by gene ontology enrichment analysis of the RNA‐sequencing data. c) CLIP‐qPCR against YTHDC1 antibody showing relative binding abundance on m^6^A sites of these factors as indicated. d) Schematic of strategy for looking for YTHDC1‐regulated factors in macrophages. e) Expression of *Rhoh* and *Nme1* in M14^−/−^ BMDMs compared to M14^f/f^ groups based on the online RNA‐sequencing data. f) Read density of m^6^A peaks in *Rhoh* and *Nme1* transcripts in M14^f/f^ and M14^−/−^ BMDMs. The m6A peaks of Nme1 were labeled by red dashed line. g) Schematic of ssRNA probes with either unmethylated or methylated adenosine (up) and western blot detection of ssRNA probes‐precipitated protein levels (bottom). h) CLIP‐qPCR against METTL14 antibody showing the relative binding abundance of m^6^A site in *Rhoh* (left) or *Nme1* (right). i) m^6^A‐IP‐qPCR showing m^6^A abundance of *Rhoh* (left) and *Nme1* (right) in M14^f/f^ and M14^−/−^ BMDMs. j) Schematic of m^6^A motif or mutant in *Rhoh* or *Nme1*. k) Luciferase reports showing the function of METL14 in wild‐type or mutated Rhoh (left) and Nme1 (right) reporters in BMDMs. l–n) *Ythdc1* ^−/−^ BMDMs transduced with or without control‐, WT *Ythdc1*‐, W378A mutant‐, or W429 mutant‐lentivirus were used for the following experiments: mRNA levels of *Rhoh* and *Nme1* tested by qPCR (l), RNA decay assays of *Rhoh* (left) and *Nme1* (right) (m), transcription rate of *Rhoh* (left) or *Nme1* (right) (n). *Ythdc1* ^f/f^ BMDMs served as controls. o,p) Western blot examinations of YTHDC1 (o) or qPCR tests (p) of *RHOH* and *NME1* in MDMs infected with or without sg*YTHDC1*‐lentivirus. q) Schematic of YTHDC1 roles in *Rhoh* and *Nme1* mRNAs. *n* = 3 for each group. Ctrl, control; ss, single strand; WT, wild type; Mut, mutant; M14, Mettl14; KO, knockout. ^**^ *p* < 0.01, ^***^ *p* < 0.001, ^****^ *p* < 0.0001 versus corresponding control group. Data depict mean ± SD. Two‐tailed Student's *t*‐test, one‐way ANOVA, and two‐way ANOVA were performed for statistical analyses.

To explore the molecular underpinning by which YTHDC1 in macrophages regulates colitis progression, we mined the published RNA‐sequencing database (GEO: GSE153512) concerning *Mettl14* ^f/f^ and *Mettl14* ^f/f^;Lysm‐Cre BMDMs since YTHDC1 depends on RNA methylation to exert its functions. Analyses showed that ≈140 genes (logFC < −1.5 or > 1.5; *p* < 0.05) were upregulated or downregulated upon *Mettl14* ablation (**Figure** **4**a). Gene ontology (GO) analysis revealed that the prevalence of gene enrichment pathways was mainly related to the regulation of inflammatory response and tissue regeneration (Figure 4b). Among the 58 candidate genes associated with immune response and tissue repair, 16 of them were found to be regulated by m^6^A modification according to the online m^6^A‐IP‐sequencing data (GEO: GSE153511). These 16 genes were then classified into two groups (inflammation‐related or epithelial‐related) and subjected to CLIP‐qPCR against YTHDC1 antibodies. Finally, two of 16, *Rhoh* and *Nme1*, were identified to be the targets of YTHDC1 (Figure 4c,d). Both expression and m^6^A peaks of *Rhoh* and *Nme1* were considerably decreased in *Mettl14* ^−/−^ macrophages (Figure 4e,f). To provide more convincing evidence, single‐stranded methylated RNA baits (ss‐m^6^A) or unmethylated controls (ss‐A) harboring the m^6^A motif of *Rhoh* or *Nme1* were used to carry out RNA pull‐down experiments (Figure 4g). As expected, YTHDC1 as well as METTL14 were selectively bound to the methylated RNA bait, but not the unmethylated controls, with a greater affinity (Figure 4g). The binding between m^6^A sites and METTL14 was also confirmed by CLIP‐qPCR (Figure 4h). m^6^A‐IP‐qPCR data showed that m^6^A abundance on *Rhoh* or *Nme1* was largely decreased upon *Mettl14* depletion (Figure 4i). Next, we inserted the fragments of *Rhoh* or *Nme1* containing wild‐type (WT) m^6^A sites or mutants into pGL3 vector to perform luciferase assays (Figure 4j). The data displayed that METTL14 overexpression significantly increased luciferase activities in reporters harboring WT *Rhoh* or *Nme1* fragment instead of mutants (Figure 4k). Accordingly, knockout of *Ythdc1* reduced mRNA expression, promoted RNA decay, and blunted transcription rate (Figure 4l–n). These events were restored by overexpression of wild‐type YTHDC1 but not mutants (Figure 4l–n). *RHOH* and *NME1* were decreased in human macrophages as well following *YTHDC1* knockout (Figure 4o,p). Together, YTHDC1 promotes *Rhoh* and *Nme1* expression in an m^6^A‐dependent way (Figure 4q).

### 2.7 RHOH deficiency in macrophages facilitates inflammatory responses

**Figure 5:**
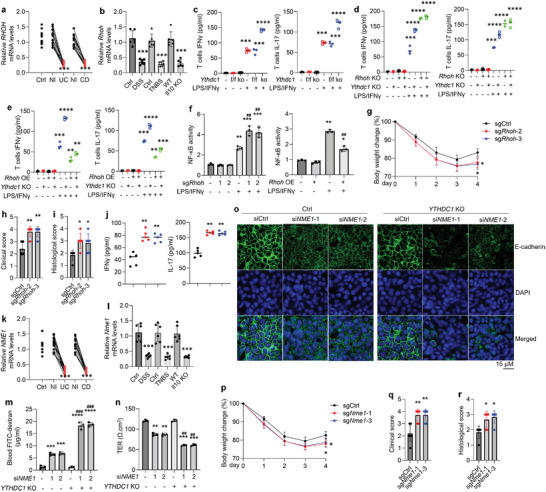
Either RHOH or NME1 is essential for maintaining the homeostasis of colon tissue in the setting of IBD. a,b) Quantitative PCR showing *RHOH*/*Rhoh* mRNA levels in MACS‐enriched gut macrophages derived from IBD patients (a) or colitis mice (b). *n* = 15 for each human group, *n* = 6 for each mouse group. c) ELISA displaying IFN‐*γ* (left) or IL‐17 (right) levels in T cells co‐cultured with *Ythdc1* ^f/f^ or *Ythdc1* ^−/−^ macrophages in the presence or absence of 8‐h LPS/IFN‐*γ* treatment. d) ELISA showing IFN‐*γ* (left) or IL‐17 (right) levels in T cells co‐cultured with or without *Ythdc1* ^−/−^/*Rhoh* ^−/−^BMDMs in the presence or absence of 8‐h LPS/IFN‐*γ* treatment. e) Elisa of IFN‐*γ* (left) or IL‐17 (right) expression in T cells co‐cultured with or without *Ythdc1* ^−/−^/*Rhoh*‐OE BMDMs in the presence or absence of 8‐h LPS/IFN‐*γ* treatment. f) NF‐*κ*B activity assays of *Rhoh*‐depleted (left) or *Rhoh*‐overexpressed (right) BMDMs with or without 8‐h LPS/IFN‐*γ* treatment. g–j) Wild‐type mice were injected with clodronate to delete endogenous macrophages and reconstituted by macrophages infected with sgCtrl‐ or sg*Rhoh*‐lentivirus prior to TNBS treatment. These assays were performed: Body weight change (g) and clinical score on day 3 post‐TNBS administration (h), *n* = 10 for each group; Histological score on day 3 post‐TNBS administration (i), *n* = 6 for each group; j) IFN‐*γ* (left) and IL‐17 (right) levels tested by ELISA in the colon mucosae on day 3 after TNBS treatment. *n* = 5 for each group. k,l) Quantitative PCR showing *NME1*/*Nme1* mRNA levels in MACS‐enriched gut macrophages derived from IBD patients (k) or colitis mice (l). *n* = 15 for each human group, *n* = 6 for each mouse group. m–o) FITC‐dextran permeability assays (m), trans‐epithelial electrical resistance measurements (n), or immunofluorescence staining (o) of siRNAs‐transfected HCT116 cells co‐cultured with or without *Ythdc1* ^−/−^BMDMs. p–r) Wild‐type mice were injected with clodronate to delete endogenous macrophages and reconstituted by macrophages infected with sgCtrl‐ or sg*Nme1*‐lentivirus prior to TNBS treatment. These assays were performed: Body weight change (p) and clinical score on day 3 post‐TNBS administration (q), *n* = 10 for each group; Histological score on day 3 post‐TNBS administration (r), *n* = 6 for each group. Ctrl, control; WT, wild type; NI, non‐inflammation; I, inflammation; UC, ulcerative colitis; CD, Crohn disease; TNBS, 2,4,6‐trinitrobenzene sulfonic acid; DSS, dextran sulfate sodium; KO, knockout; TER, trans‐epithelial electrical resistance. *p < 0.05, ^**^ *p* < 0.01, ^***^ *p* < 0.001, ^****^ *p* < 0.0001 versus corresponding control group; ^##^ *p* < 0.01, ^###^ *p* < 0.001 versus sg*Rhoh*, *Rhoh*‐OE, or si*NME1* alone group. Data depict mean ± SD. Two‐tailed Student's *t*‐test, one‐way ANOVA, and two‐way ANOVA were performed for statistical analyses.

To explain the impact of YTHDC1 on macrophage polarization, we treated *Ythdc1* ^f/f^ or *Ythdc1* ^−/−^ macrophages with LPS/IFN*γ* or IL‐4/IL‐13. Interestingly, the lack of YTHDC1 promoted M1 macrophage polarization but had no influence on that of M2 (Figure S6a–f, Supporting Information). RHOH, which showed dramatic decreases in the macrophages from diseased tissues (**Figure** **5**a,b; Figure S6l,m, Supporting Information), is referred to be closely associated with T cell differentiation.^[^ ^20^ ^]^ To this end, we deleted RHOH and tested its functions in macrophages (Figure S6g, Supporting Information). As shown, elimination of RHOH contributed to M1 macrophage‐related cytokines production (Figure S6h, Supporting Information). We next established a macrophage‐T cell co‐culture system to dissect the roles of macrophages with *Ythdc1* deletion in T cells under M1 polarization conditions (Figure S6i, Supporting Information). Of note, *Ythdc1* knockout favored IFN‐*γ* and IL‐17 expression in T cells co‐cultured with macrophages in the presence of LPS/IFN‐*γ* (Figure 5c). Meanwhile, *Rhoh* knockout in macrophages replicated the phenotypes observed upon *Ythdc1* deletion (Figure 5d). Reversely, forced expression of RHOH in macrophages inhibited cytokines production in the setting of M1 polarization (Figure S6j, Supporting Information, and Figure 5e). These new findings indicate that RHOH is able to suppress inflammatory responses in macrophages and subsequently inactivate T cells differentiation. In line with other studies noting RHOH inhibits the activation of nuclear factor kappa B (NF‐*κ*B) pathway in T cells,^[^ ^20a^ ^]^ our results delineated that RHOH could block NF‐*κ*B activities in macrophages following LPS/IFN‐*γ* challenge (Figure 5f), implying inactivation of the NF‐*κ*B pathway is likely to be the molecular mechanism by which RHOH regulates inflammatory responses in macrophages. What is more, the deletion of *Rhoh* in macrophages boosted colitis development in the animal model (Figure 5g–j and Figure S6k, Supporting Information).

### 2.8 The Lack of NME1 in Macrophages Compromises Colonic Epithelial Barrier Integrity

To interrogate the functions of NME1 in colon tissues, we purified or cultured colonic epithelial cells, macrophages, T cells, B cells, dendritic cells, neutrophils, and fibroblasts for analyses. As shown, NME1 was highly expressed in colonic epithelial cells and macrophages (Figure S7a,b, Supporting Information). NME1, which showed robust decreases in the macrophages and colonic epithelial cells from diseased tissues (Figure 5k,l; Figures S6n,o and S7c,d, Supporting Information), is identified to be required for maintaining the adherens junctions of colon carcinoma cells.^[^ ^21^ ^]^ Recently, NME1 is reported to be a member of the macrophage secretomes that are secreted to exert biological actions outside of macrophages.^[^ ^22^ ^]^ Thus, we reasoned that macrophage‐produced NME1 might be a main source compensating for the loss of that secreted from colonic epithelial cells. To validate this hypothesis, we knocked down *NME1* in colonic epithelial cells and co‐cultured them with wild‐type or *YTHDC1* knockout macrophages using transwell inserts. *NME1* knockdown did not influence cell growth in colonic epithelial cells (Figure S7e,f, Supporting Information). Consistent with other studies, NME1 deficiency increased FITC‐dextran paracellular infiltration, decreased the transepithelial electrical resistance (TER), and destroyed intercellular adhesion of colonic epithelial cell monolayers (Figure 5m–o). However, in the co‐culture system, macrophages with *YTHDC1* deletion accelerated the impaired integrity of colonic epithelial cell monolayers compared to control groups (Figure 5m–o). Similar results were also observed in the co‐culture system using macrophages with or without *NME1* depletion (Figure S7g–j, Supporting Information). To confirm NME1 in the macrophage culture media is indispensable for the colonic epithelial cell monolayers, we immuno‐depleted NME1 from macrophage media using anti‐NME1 antibodies (Figure S7k, Supporting Information) and mixed them with DMEM media (v/v, 50%/50%) for colonic epithelialc cells (CEC) culture. Likewise, macrophage media without NME1 facilitated monolayers damage in contrast to those with NME1 (Figure S7l–n, Supporting Information), indicating the importance of extracellular NME1 for adhesion. Moreover, mice carrying *Nme1*‐depleted macrophages showed more severe colitis and greater impairment of the epithelial layers (Figure 5p–r and Figure S7o–q, Supporting Information). These data, in total, suggest that NME1 produced by macrophages enhances the integrity of colonic epithelial barrier.

### 2.9 Overexpression of Either *Rhoh* or *Nme1* in Macrophages Slowed Colitis Development

To explore whether macrophages overexpressing *Rhoh* or *Nme1* could protect mice from developing colitis, we infected BMDMs with *Rhoh*‐ or *Nme1*‐lentivirus (Figures S6j and S8a, Supporting Information). As anticipated, overexpression of either *Rhoh* or *Nme1* could rescue the loss of body weight, decrease clinical symptoms, and reduce colon tissue damage and cytokines levels (Figure S8b–f, Supporting Information).

### 2.10 YTHDC1 Plays Its Regulatory Roles in *Rhoh* and *Nme1* in the Nucleus

**Figure 6:**
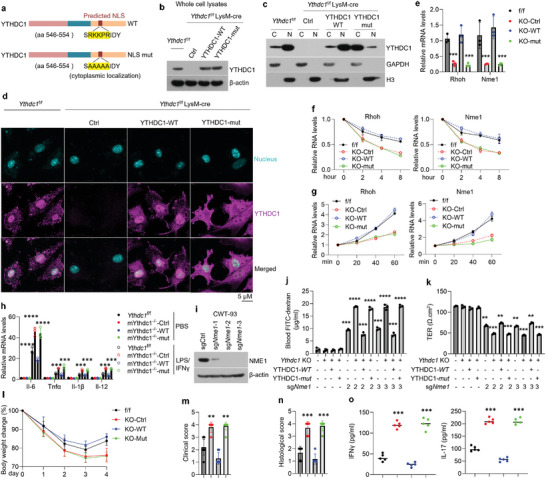
YTHDC1 exerts biological functions in the nucleus. a) Schematic of YTHDC1 (WT) and exclusively cytoplasmic YTHDC1 (NLS mut). b–h) *Ythdc1* ^−/−^BMDMs were transduced with control‐, WT *Ythdc1*‐, or NLS mutant‐lentivirus for the following experiments, *Ythdc1* ^f/f^ BMDMs were served as controls: Western blot showing YTHDC1 expression in the whole cell lysates (b) or cytoplasmic and nuclear fractions (c); Immunofluorescence staining exhibiting the location of YTHDC1 in BMDMs (d); *Rhoh* and *Nme1* levels detected by qPCR (e); RNA decay (f) and transcription rate (g) of *Rhoh* and *Nme1*; M1 macrophage‐related cytokines levels in BMDMs with or without 8‐h LPS/IFN‐*γ* treatment (h). *n* = 3 for each group. i) NME1 expression in CWT‐93 cells with or without sg*Nme1*‐lentivirus infection. *n* = 3 for each group. j–k) FITC‐dextran permeability assays (j), trans‐epithelial electrical resistance measurements (k) of *Nme1*‐deleted CWT‐93 cells co‐cultured with or without *Ythdc1* ^−/−^BMDMs in the presence or absence of WT/NSL‐mut YTHDC1 overexpression. *n* = 3 for each group. l–o) Wild‐type mice were injected with clodronate to delete endogenous macrophages and were reconstituted by macrophages infected with *Ythdc1* ^f/f^ BMDMs or *Ythdc1* ^−/−^ BMDMs transduced with control‐ or WT/NSL‐mut YTHDC1‐lentivirus prior to TNBS treatment. These assays were performed: Body weight change (l) and clinical score on day 3 post‐TNBS administration (m), *n* = 10 for each group; Histological score on day 3 post‐TNBS administration (n), *n* = 6 for each group; IFN‐*γ* and IL‐17 levels tested by ELISA in the mucosae of mice on day 3 post‐TNBS administration (o), *n* = 5 for each group. aa, amino acid; Ctrl, control; NLS, nuclear localization signal; C, cytoplasm; N, nucleus; KO, knockout; WT, wild type; Mut, mutant; TER, trans‐epithelial electrical resistance measurements. *p < 0.05, ^**^ *p* < 0.01, ^***^ *p* < 0.001, ^****^ *p* < 0.0001 versus corresponding control group. Data depict mean ± SD. One‐way ANOVA and two‐way ANOVA were performed for statistical analyses.

Since YTHDC1 is referred to reside in the nucleus mainly,^[^ ^1a^ ^]^ we are intrigued where YTHDC1 mediates *Rhoh* and *Nme1* expression in macrophages. We next generated a YTHDC1 mutant expressed in the cytoplasm exclusively by changing the canonical nuclear localization signal (NLS) (**Figure** **6**a). As shown, this mutation did not affect YTHDC1 protein levels in whole cell lysates (Figure 6b). However, wild‐type YTHDC1 was primarily located in nucleus while mutant is mostly restricted in cytoplasm (Figure 6c,d). Functionally, the YTHDC1 mutant failed to stop *Rhoh* and *Nme1* decreases, limit RNA decay and increase transcription rate compared to wild‐type groups (Figure 6e–g). Likewise, YTHDC1 mutant could not suppress inflammatory responses and strengthen integrity of monolayers formed by colonic epithelial cells (Figure 6h–k), indicating that YTHDC1 exerts its biological functions in the nucleus. We further confirmed that YTHDC1 mutant could not reverse body weight loss, clinical/histological scores, and IFN‐*γ* and IL‐17 increases in the TNBS‐induced colitis model (Figure 6l–o).

**Figure 7:**
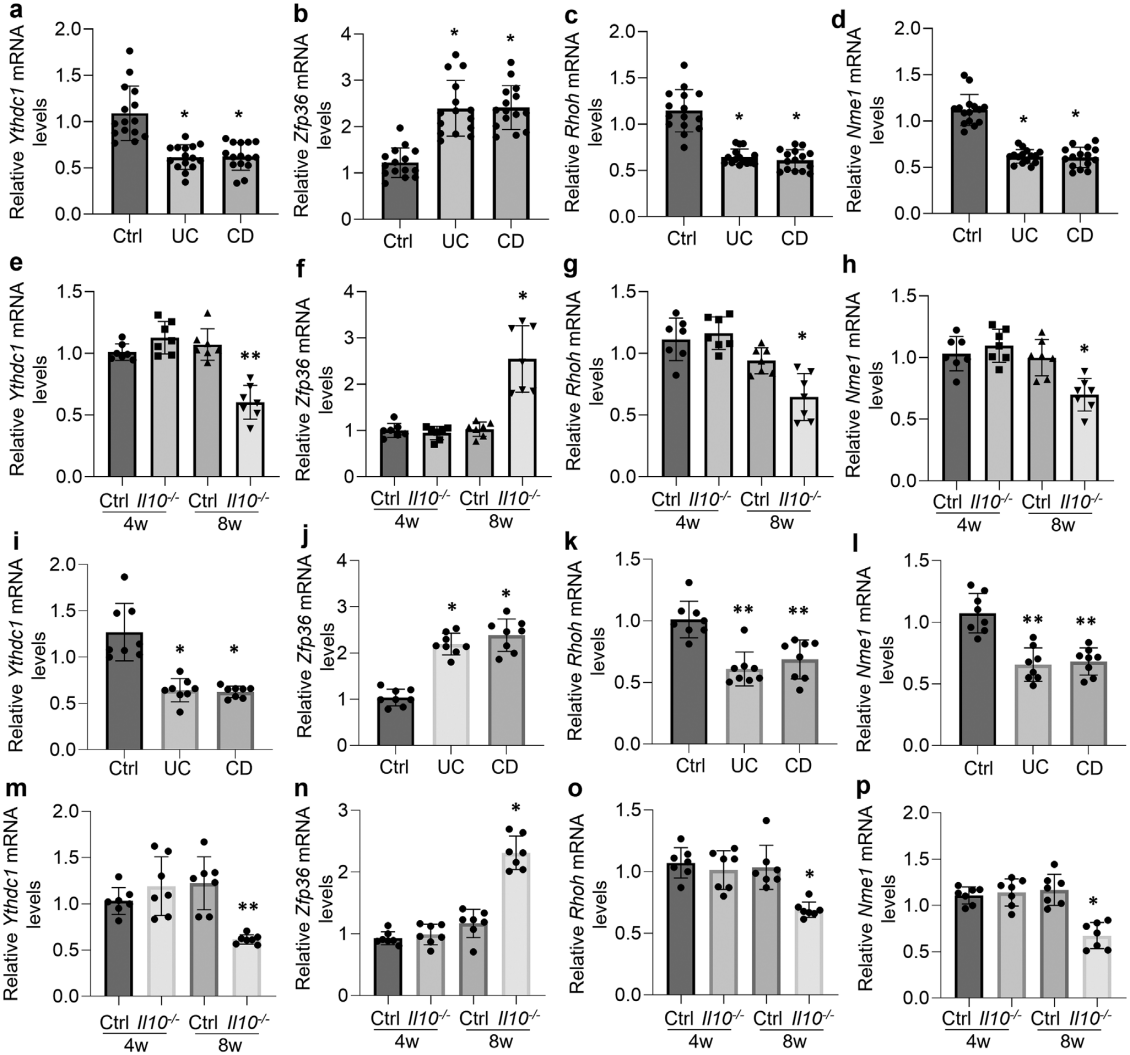
IBD‐derived microbiotas decrease YTHDC1 levels in gut macrophages. a–d) Quantitative PCR showing *Ythdc1* (a), *Zfp36* (b), *Rhoh* (c), or *Nme1* (d) mRNA levels in MACS‐enriched gut macrophages from wild type mice transplanted with microbiotas from healthy individuals or IBD patients. *n* = 15 for each group. e–h) Quantitative PCR showing *Ythdc1* (e), *Zfp36* (f), *Rhoh* (g), or *Nme1* (h) mRNA levels in MACS‐enriched gut macrophages from wild‐type mice transplanted with microbiotas from 4‐ or 8‐week‐old wild type/*Il10* ^−/−^ mice. *n* = 7 for each group. i–l) Quantitative PCR showing *Ythdc1* (i), *Zfp36* (j), *Rhoh* (k), or *Nme1* (l) mRNA levels in FACS‐enriched gut macrophages from wild‐type mice transplanted with microbiotas from healthy individuals or IBD patients. *n* = 8 for each group. m–p) Quantitative PCR showing *Ythdc1* (m), *Zfp36* (n), *Rhoh* (o), or *Nme1* (p) mRNA levels in FACS‐enriched gut macrophages from wild‐type mice transplanted with microbiotas from 4‐ or 8‐week‐old wild‐type/*Il10* ^−/−^ mice. *n* = 7 for each group. Ctrl, control; UC, ulcerative colitis; CD, Crohn disease; 4w, 4 weeks; 8w, 8 weeks. *p < 0.05, ^**^ *p* < 0.01 versus corresponding control group. Data depict mean ± SD. One‐way ANOVA was performed for statistical analyses.

Fecal microbiotas from IBD patients and *Il10* ^−/−^ mice developing colitis decrease gut macrophage YTHDC1 expression in wild‐type mice. Microbiota is identified to affect the progression and development of IBD which is correlated with altered gut microbiota.^[^ ^23^ ^]^ We next aimed to decipher whether the altered microbiota from IBD could influence YTHDC1 expression in intestinal macrophages. Wild‐type mice were treated with antibiotics for 3 days to eradicate the original microbiotas and then underwent fecal microbiota transplantation (FMT) with microbiotas from IBD patients (Figure S9a, Supporting Information). Importantly, wild‐type mice who received microbiotas from IBD patients showed decreases of *Ythdc1*, *Rhoh*, and *Nme1* and increases of *Zfp36* in gut macrophages compared to those colonized with microbiotas from healthy volunteers (**Figure** **7**a–d). Colonization of human microbiotas was confirmed by testing the relative abundances of altered bacteria species (Figure S9b, Supporting Information). In contrast to wild‐type mice, the compositions of microbiotas in *Il10* ^−/−^ mice are also changed.^[^ ^24^ ^]^ In agreement with the notion that *Il10* ^−/−^ mice would not develop colitis until 2 months,^[^ ^15a^ ^]^ we observed evident rectal prolapse in 8‐week‐old *Il10* ^−/−^ mice but not in 4‐week‐old ones (Figure S9c, Supporting Information). Consistently, colitis development and microbiota dysbiosis were also observed in 8‐week‐old *Il10* ^−/−^ mice (Figure S9d–f, Supporting Information). Wild‐type mice who received microbiotas from 8‐week‐old *Il10* ^−/−^ mice exhibited reductions of *Ythdc1*, *Rhoh*, and *Nme1* and increases of *Zfp36* in gut macrophages in contrast to those transplanted with microbiotas from 4‐week‐old *Il10* ^−/−^ mice (Figure 7e–h). These findings were also confirmed in FACS‐enriched macrophages (Figure 7i–p). Together, these data demonstrate that fecal microbiotas from IBD patients or colitis mice could downregulate YTHDC1 levels in gut macrophages.

## 3 Discussion

In this study, we tested the functions of YTHDC1 in macrophages under IBD conditions by establishing both chemically induced and spontaneous colitis models using the *Ythdc1* ^f/f^ and m*Ythdc1* ^−/−^ mice. According to the literature, there are three major categories of IBD animal models: those which are induced chemically, those which are triggered by adoptive CD4^+^CD45RB^hi^ T Cells transferred into Rag^−/−^ mice, and those which develop colitis spontaneously in mice with genetical modification such as *Il10* ^−/−^ animals.^[^ ^25^ ^]^ There is no single colitis model that thoroughly recapitulates the histopathological and clinical features of human IBD, therefore, the choice of IBD models mainly depends on the cell type being targeted for investigation or therapy.^[^ ^26^ ^]^ Colitis models induced by chemicals such as DSS and TNBS are the most widely used. DSS administration leads to severe colitis showing weight loss, bloody diarrhea, epithelial cell damage, ulcer formation, and neutrophil infiltrations, mimicking some characteristics of flares in human UC. Administration of TNBS, a haptenating agent, causes an immune response and results in transmural colitis representing clinical Crohn's disease.^[^ ^27^ ^]^ Here, we confirmed that gut macrophages lacking YTHDC1 benefit colitis progression in both DSS‐ and TNBS‐induced animals, revealing the requirement of YTHDC1 for limiting IBD development. Given that monocytes are the main source of IL‐10 production,^[^ ^28^ ^]^ we used *Il10* ^−/−^ mice developing colitis spontaneously for further investigation. Since T cells are not the main point of this project, the adoptive CD4^+^CD45RB^hi^ T Cell transfer mouse colitis model was not involved.

Macrophages, the innate immune cells, are reported to be closely associated with the initiation and development of IBD.^[^ ^14^, ^29^ ^]^ Macrophages in tissues are developed from monocytes circulating throughout the blood.^[^ ^30^ ^]^ In the mammalian intestinal tract, tissue‐resident macrophages are indispensable for sustaining gut homeostasis and function.^[^ ^14^ ^]^ Intestinal‐resident macrophages exhibiting high plasticity perpetuate inflammatory responses in IBD via releasing excessive inflammatory mediators. In addition to forming a defense line against pathogens, tissue‐resident macrophages promote tissue repair and regeneration following injury.^[^ ^29^ ^]^ Herein, we pointed out YTHDC1 expression in macrophages derived from IBD or colitis samples is decreased and macrophage‐specific deficiency of YTHDC1 accelerates disease development in animal colitis models. Consistent with the two major aspects of macrophages’ effects on IBD development mentioned above, we provided convincing data indicating YTHDC1 regulates *Rhoh* to counteract overwhelming inflammatory response and mediates *Nme1* to enhance colonic epithelial barrier formation. RHOH is known to be a potent inhibitor of NF‐*κ*B activation and influence T cell differentiation.^[^ ^20a^ ^]^ Similarly, we also found that RHOH can inactivate NF‐*κ*B activity to block M1 macrophage polarization. M1 macrophages are pro‐inflammatory and secrete cytokines such as TNF‐*α*, IL‐1*β*, IL‐6, IL‐12, and IL‐23 to regulate the differentiation of T cells.^[^ ^31^ ^]^ Therefore, RHOH could regulate pro‐inflammatory cytokines secretion in macrophages to affect the differentiation of both Th1 and Th17 T cells. Moreover, the loss of macrophage‐produced NME1 weakens the integrity of monolayers formed by CEC with deficient NME1, in line with the view that NME1 silencing disrupts cell–cell adhesion of human colon cancer cells.^[^ ^21^ ^]^

Macrophage depletion and reconstitution system is well established to explore the in vivo functions of macrophages.^[^ ^19^ ^]^ In this system, mice were injected with clodronate‐liposomes to erase the endogenous macrophages, followed by another injection with in vitro lentivirus‐transduced and well‐differentiated BMDMs for reinstitution. This is a good tool for the myeloid cell‐specific gene deletion or overexpression studies in mice. However, the major downside of this system is that the endogenous macrophages in mice would be replenished in one week. To this end, only the acute TNBS‐induced colitis animal model is qualified for this system. Although we provided compelling evidence using this macrophage depletion and reconstitution system, more approaches such as the genetically engineered mouse model are warranted to confirm RHOH and NME1 in macrophages are potential therapeutic targets for IBD management.

ZFP36, also termed as tristetraprolin (TTP), is able to regulate inflammatory responses.^[^ ^32^ ^]^ We reported here that increased ZFP36 in macrophages accounts for YTHDC1 decrease. Interestingly, other studies have suggested a high expression of ZFP36 in IBD‐derived macrophages compared to healthy donors.^[^ ^33^ ^]^ Published data state that ZFP36 and TNF*α* are positively correlated in IBD‐derived macrophages,^[^ ^33^ ^]^ but the mechanism regarding this correlation is unclear. Based on our current results, we explain that upregulation of cytokines in IBD‐derived macrophage depends on, at least in part, the ZFP36–YTHDC1–RHOH axis.

Recently, pioneering research has suggested the communication between gut microbiota and macrophages is important for maintaining intestinal homeostasis.^[^ ^29^ ^]^ Particular attentions are now focusing on microbiota‐produced metabolites, believed to have immunomodulatory abilities to manipulate macrophage plasticity.^[^ ^29^ ^]^ Here, we reported that microbiotas from IBD patients or mice are capable of decreasing YTHDC1 expression in intestinal‐resident macrophages. We reasoned that microbiota‐derived metabolites might be responsible for the downregulation of YTHDC1 in macrophages, but this hypothesis needs to be verified in the future.

In conclusion, this study highlights a previously unrecognized role of macrophage‐specific YTHDC1 in mediating IBD development. Since YTHDC1 is necessary for maintaining embryonic stem cell proliferation,^[^ ^6a^ ^]^ relative investigations concerning YTHDC1 and intestinal stem cells would be very interesting.

## 4 Experimental Section

### Human Subjects

Surgically resected colon tissues and colonic mucosal biopsies were harvested and processed as described.^[^ ^34^ ^]^ Fresh human blood samples were collected from healthy individuals. All human samples were received from the Second Hospital of Shanxi Medical University. Colonic specimens were obtained from evident macroscopic inflammation areas, and normal‐appearing mucosa (non‐inflammation), accompanied by confirmed diagnoses of CD or UC by histopathologic examinations. Healthy control samples were from patients who underwent therapeutic bowel resection for malignant or non‐malignant, non‐inflammatory conditions and from those who underwent screening colonoscopy without endoscopic and histopathological colonic mucosal disorders. This project was approved by the Ethics Committee of the Second Hospital of Shanxi Medical University (#2022YX110) and informed consent was signed by all subjects. More details on participants were provided in Table S1 (Supporting Information).

More details are presented in Supporting Information.

## Conflict of Interest

The authors declare no conflict of interest.

## Author contributions

X.G., G.X., Y.D., R.L., and K.H. contributed equally to this work. J.D. conceived and designed the research. X.G., G.X., Y.D., R.L., and T.X. performed the experiments. B.Z. provided assistant work for experiments. G.X. collected human samples. C.W., K.H., W.L., and M.S. analyzed data. C.W. supervised the patient research. J.D. wrote the manuscript. J.D. and Y.D. acquired funding. All authors read and approved the final manuscript.

## Supporting information

Supporting Information

## Data Availability

The data that support the findings of this study are available from the corresponding author upon reasonable request.
