# Supplementary material for: The Loss of YTHDC1 in Gut Macrophages Exacerbates Inflammatory Bowel Disease
Source: Adv Sci (Weinh). 2023 Mar 15;10(14):2205620. doi: 10.1002/advs.202205620 (PMC10190588; doi:10.1002/advs.202205620)
Supplement: Supplementary file 1 — Supporting Information [file ADVS-10-2205620-s001.pdf]

The loss of YTHDC1 in gut macrophages exacerbates inflammatory bowel disease

Xuejun Ge[1,10], Gang Xue[2,10], Yan Ding[3,4,10], Ran Li[1,10], Kaining Hu[5,10], Tengjiao Xu[4], Ming Sun[6], Wang Liao[7], Bin Zhao[1], Chuangyu Wen[8]*, Jie Du[1,9]*

1 Shanxi Province Key Laboratory of Oral Diseases Prevention and New Materials, Shanxi Medical University School and Hospital of Stomatology, Taiyuan, Shanxi, China.

2 Department of Gastroenterology, Second Hospital of Shanxi Medical University, Taiyuan, Shanxi, China

3 Department of Dermatology, Hainan Provincial Hospital of Skin Disease, Haikou, Hainan, China.

4 Department of Dermatology, Hainan Medical University Affiliated Dermatology Hospital of Hainan Medical College, Haikou, Hainan, China.

5 Department of Human Genetics, The University of Chicago, Chicago, IL, USA

6 College of Life Sciences, Mudanjiang Medical University, Mudanjiang, Heilongjiang, China.

7 Department of Cardiology, Hainan General Hospital and Hainan Affiliated Hospital of Hainan Medical University, Haikou, China.

8 Central Laboratory, Affiliated Dongguan Hospital, Southern Medical University, Dongguan, Guangdong, China.

9 Institute of Biomedical Research, Shanxi Medical University, Taiyuan, Shanxi, China.

10 These authors contributed equally

Supplemental table1-2

Supplemental figure1-9

Methods and materials

Table S1

Details of IBD patients and healthy controls

Subtype	NO.	Gender	Age	Sites	Inflammatory condition
UC	1	Male	39	Colon	Active
UC	2	Male	35	Colon	Active
UC	3	Male	36	Colon	Active
UC	4	Male	56	Colon	Active
UC	5	Female	29	Colon	Active
UC	6	Male	31	Colon	Active
UC	7	Male	66	Colon	Active
UC	8	Male	60	Colon	Active
UC	9	Female	70	Colon	Active
UC	10	Female	60	Colon	Active
UC	11	Male	48	Colon	Active
UC	12	Male	34	Colon	Active
UC	13	Male	60	Colon	Active
UC	14	Female	55	Colon	Active
UC	15	Female	30	Colon	Active
CD	1	Male	60	Colon	Active
CD	2	Male	55	Colon	Active
CD	3	Male	37	Colon	Active
CD	4	Male	47	Colon	Active
CD	5	Female	55	Colon	Active
CD	6	Female	59	Colon	Active
CD	7	Female	39	Colon	Active
CD	8	Female	71	Colon	Active
CD	9	Male	67	Colon	Active
CD	10	Male	21	Colon	Active
CD	11	Male	38	Colon	Active
CD	12	Female	47	Colon	Active
CD	13	Male	60	Colon	Active
CD	14	Male	31	Colon	Active
CD	15	Male	29	Colon	Active
HC	1	Female	25	Colon	None
HC	2	Male	54	Colon	None
HC	3	Male	73	Colon	None
HC	4	Male	72	Colon	None
HC	5	Male	36	Colon	None
HC	6	Male	77	Colon	None
HC	7	Female	36	Colon	None
HC	8	Female	26	Colon	None
HC	9	Female	76	Colon	None
HC	10	Female	42	Colon	None

HC	11	Female	32	Colon	None
HC	12	Male	66	Colon	None
HC	13	Male	30	Colon	None
HC	14	Male	21	Colon	None
HC	15	Male	51	Colon	None

CD, Crohn’s disease; UC, ulcerative colitis; HC, healthy controls

Table S2

Sequences of primers involved in the study

**RT-PCR primers**		
Name	Forward 5'-3'	Reverse 5'-3'
mF4/80	CTGCACCTGTAAACGAGGCTT	GCAGACTGAGTTAGGACCACAA
mCd11b	CCATGACCTTCCAAGAGAATGC	ACCGGCTTGTGCTGTAGTC
mBrf1	CCTGGACACAGCATTCAATTTCT	TTCGGCAAACCAAGTAGAGGC
mKhsrp	ACTGGAGCACCTGAGTCTGT	CGTTGTCGTGAAACTGTCCT
mHnrnpd	AGTCGGAGAGTGTAGATAAGGTC	GGCCCTTTTAGGATCAATGACTT
mTia1	CAGTGGCTTGGTGGAAGACAA	TGGGTCTGACGAACAAATGAG
mTial1	TGATAACAGAGCAACCCGATAGC	TTCCACAAAGCAATATGGGTCAT
mZfp36	TCTCTGCCATCTACGAGAGCC	TCCTCCGAGGGATTCGGTTC
mRhoh	AACCCACGGTGTACGAGAATA	GGGCCGGATACTTCTGAAGG
mNme1	AGGAGCACTACACTGACCTGA	GGTTGGTCTCTCCAAGCATCA
mIl-6	CCTCTCTGCAAGAGACTTCCA	AGAATTGCCATTGCACAACTCT
mTnfα	TCAGCCTCTTCTCATTCCTG	CAGGCTTGTCACTCGAATTT
mIl-1β	CCAAAAGATGAAGGGCTGCT	ACAGAGGATGGGCTCTTCTT
mIl-12	CAATCACGCTACCTCCTCTTTT	CAGCAGTGCAGGAATAATGTTTC
mIl-17	TCAGCGTGTCCAAACACTGAG	CGCCAAGGGAGTTAAAGACTT
mIl-23	CAGCAGCTCTCTCGGAATCTC	TGGATACGGGGCACATTATTTTT
mF4/80	CTGCACCTGTAAACGAGGCTT	GCAGACTGAGTTAGGACCACAA
mMac-1	CCATGACCTTCCAAGAGAATGC	ACCGGCTTGTGCTGTAGTC
mIl-10	CTTACTGACTGGCATGAGGATCA	GCAGCTCTAGGAGCATGTGG
mIfn-γ	GCGTCATTGAATCACACCTG	TGAGCTCATTGAATGCTTGG
mArg1	CTCCAAGCCAAAGTCCTTAGAG	GGAGCTGTCATTAGGGACATCA
mFizz1	CCAATCCAGCTAACTATCCCTCC	ACCCAGTAGCAGTCATCCCA
mYm1	CAGGTCTGGCAATTCTTCTGAA	GTCTTGCTCATGTGTGTAAGTGA
mYthdc1	GAGAATGGAGTCTACTGACACCA	ACAGACGAATTTTTCGATCAGCA
mGapdh	AGGTCGGTGTGAACGGATTTG	TGTAGACCATGTAGTTGAGGTCA
hCD14	ACGCCAGAACCTTGTGAGC	GCATGGATCTCCACCTCTACTG
hCD11B	GCCTTGACCTTATGTCATGGG	CCTGTGCTGTAGTCGCACT
hBRF1	GGTGTGCCTCACGCATCTC	GAAGGCCCCTCATCCTACAGA
hKHSRP	ATCCGCAAGGACGCTTTCG	TGCTCTCCGGTTGATCTCCAT
hHNRNPD	GCGTGGGTTCTGCTTTATTACC	TTGCTGATATTGTTCCTTCGACA
hTIA1	CGAGATGCCCAAGACTCTATAC	CCTTACCCATTATCTTCCGTCCA
hTIAL1	TGGTTGGGTGGTCGTCAAATC	CAGACGCAATTCCTCCACAGT
hZFP36	GACTGAGCTATGTCGGACCTT	GAGTTCCGTCTTGTATTTGGGG
hYTHDC1	AACTGGTTTCTAAGCCACTGAGC	GGAGGCACTACTTGATAGACGA
hRHOH	ATGCTGAGTTCCATCAAGTGC	TCTGCCTGCTGGTAGGACA
hNME1	AAGGAGATCGGCTTGTGGTTT	CTGAGCACAGCTCGTGTAATC
hIL-6	ACTCACCTCTTCAGAACGAATTG	CCATCTTTGGAAGGTTCAGGTTG
hTNFα	CCTCTCTCTAATCAGCCCTCTG	GAGGACCTGGGAGTAGATGAG
hIL-1β	ATGATGGCTTATTACAGTGGCAA	GTCGGAGATTCGTAGCTGGA

hIL-12	CCTTGCACTTCTGAAGAGATTGA	ACAGGGCCATCATAAAAGAGGT
hIL-23	CTCAGGGACAACAGTCAGTTC	ACAGGGCTATCAGGGAGCA
hGAPDH	GGAGCGAGATCCCTCCAAAAT	GGCTGTTGTCATACTTCTCATGG
**Bacteria PCR**		
Name	Forward 5'-3'	Reverse 5'-3'
Bacteroides acidifaciens	CAGCATGAAAGTTTGCTTGCAA	CCATCATGCGGTAGGACTATGA
Helicobacter hepaticus	GAGCCTCAAAAGGTTTATAGC	CTATTTCATATCCATAAGCTCTTGA GAATC
Lactobacillus murinus	GCAATAGCCATGCAGCTATTGTTG	GCAATGATGCGTAGCCGAAC
Firmicutes	TGAAACTYAAGGAATTGACG	ACCATGCACCTGTC
Lachnospiraceae	TTCGCAAGAATGAAACTCAAAG	AAGGAAAGATCCGGTTAAGGATC
Bifidobacterium longum	ACCATCTGGGTGGAGAAAGTG	TGGCGGAAATGAACTCGTAAT
Eubacterium rectale	CGGTACCTGACTAAGAAGC	AGTTTCATTCTTGCGAAC
Faecalibacterium prausnitzii	CCATGAATTGCCTTCAAAACTGTT	GAGCCTCAGCGTCAGTTGGT
Blastocystis	GGAGGTAGTGACAATAAATC	TGCTTTCGCACTTGTTCATC
Bacteroides fragilis	TGAATACATTTCCTTTTTGCCTCT	CCTACACCTTCCTTGTATATCTCCA T
Ruminococcus torques	GACGGTAATGCGTCCTTCC	TGGCCGCTGGCTACTAAAG
Clostridium hathewayi	GGAGCGTAGACGGTTTAG	CGGTTAAAGTGTTTTCAGTG
Clostridium bolteae	CCTCTTGACCGGCGTGT	CCTAGAGTGCCCAGCTTTACCTG
16S rRNA	TACCTTGTTACGACTT	AGAGTTTGATCMTGGCTCAG
**RIP-qPCR and CLIP-qPCR primers**		
Target site	Forward 5'-3'	Reverse 5'-3'
hYTHDC1 AU-rich site	AGATACACAGAGTTATGTTA	ACAGAAGACTAAAACAACAA
mYthdc1 AU-rich site	AAGCACTGACTGAAGATAAA	ACTGTGCATTTTTTTGGCGG
Serpine1 IP-seq	TTGGAAAGATTACCAGTGTGGGG	ATGGGCCAAGTGATGGAGCCTTG AC
Il17ra IP-seq	TTCAACATCACCTCCAGGTACCC AC	CTGCCGGCCACTAGGGCTCTG
C1qa IP-seq	CAGTGCCCTGGTAAATGCGACCC T	CCAGGGATTCCCTGAGTTTCTCTA A
Apoe IP-seq	CCCTTGAGGCGGGCCTGGAAGAT CTCC	AGTGGGCAACCAGGCCCGTGACC G

Plau IP-seq	AAATAAATGATTTCCCAATTAGGAAGT	TTCCTGATCCATTCATGGGATGCTAGAG
Pdpn IP-seq	TCTTAACAGTAATAGGGACTGGCC	ATAAGAGAGCTTCCAACTTGCCCTGT
Sdc1 IP-seq	ACTCTTCTGACTTGGTTTCTCCAAAT	CTAAAGTAGACCTCCCCACAATGAAA
Anxa2 IP-seq	GCAAGACACCAAGGGTGACTACC	AGGAGAGAATGAGGAGAAAGCCAGGCAA
B4galt1 IP-seq	GACTGTACAAATGAGAGGGACCAGC	TCGCACATACAAAGGAAACGATGCG
Gpnmb IP-seq	TAGGAAACTGCCCCAGGAACACGGT	TTCCCTCTGACCAGGAACCCACTCTTCT
Rhoh IP-seq	ATTGAGCCCTTGAGGGTTCTTTCC	CACCAACAGTGAGGTTTTCCCCAC
Runx3 IP-seq	CCTGAGCACGCCGGGCCGCATGGAC	CAGCTAGAGAGGACATTGATTTGGGTT
Rara IP-seq	TGGAGATCCCAGGCTCCATGCCACC	GTCATGGGGATTGGGTGGCTGGGC
Il6ra IP-seq	ATCTGGGGAATAAGTAGTCTCTG	GCCCAAGGAATACGGTGGGGGTG
Nme1 IP-seq	ACAAGAGCTGTGCGCAGAACTGGA	AGTCACAGCTCCCAAAGGTTCCTTCC
Ctsl IP-seq	GTCAGTTTTAAGCTGAATTCCTTTG	GGAAGTGAATGGGGTATGGAAGGCT
**Lentivirus and plasmids construction primers**		
Name	Forward 5'-3'	Reverse 5'-3'
pGL3-YTHDC1	AGATACACAGAGTTATGTTA	ACAGAAGACTAAAACAACAA
pGL3-Ythdc1	AAGCACTGACTGAAGATAAA	ACTGTGCATTTTTTTGGCGG
pGL3-Rhoh	ATTGAGCCCTTGAGGGTTCT	CACCAACAGTGAGGTTTTCCC
pGL3-Nme1	ACAAGAGCTGTGCGCAGAACT	AGTCACAGCTCCCAAAGGTTCC
mYthdc1 cDNA	ATGGCGGCCGACAGCCGGGAG	TTATCTTCGATAACGACCTCT
mRhoh cDNA	ATGCTGAGCTCAATCAAGTGCG	TTAGAAGATCTTGCATTCATTGA
mNme1 cDNA	ATGGCCAACAGTGAGCGCACCT	TCACTCATAGATCCAGTTCTGCG
mMettl14 cDNA	ATGGATAGCCGCCTGCAGGAGA	GATGGCTCCTCATTTCGGCGGAG
**sgRNA sequences**		
sgZfp36-1	GAGCTCGGTCTTGTATCGAG	
sgZfp36-2	AACTCGGACTCCATCCCGTC	
sgZfp36-3	GGATCTCTCTGCCATCTACG	
sgZFP36-1	GTGCCCGTGCCATCCGACCA	
sgZFP36-2	GAGAGTGGGCGCTGCCGCTA	
sgZFP36-3	CATGGCCAACCGTTACACCA	
sgNme1-1	CAGAAGGGGTTCCGCCTTGT	
sgNme1-2	AAGTCTCCTCGTATGGTCCC	
sgNme1-3	AAGGACCGCCCCTTCTTTAC	

sgNME1-1	ATCTGGTTTGATCGCAATGA
sgNME1-2	TTCATTGCGATCAAACCAGA
sgNME1-3	CAAAGAATGGACGGTCCTTC
sgYthdc1-1	TGATTATGACACCCGAAGTG
sgYthdc1-2	AGATGGGGAACTTAATGTTT
sgYthdc1-3	TACAATTCATCATCCTGTTC
sgYTHDC1-1	GAGCCATGGCGGCTGACAGT
sgYTHDC1-2	AGATGGAGAACTTAATGTTC
sgYTHDC1-3	CTCCCGACTGTCAGCCGCCA
sgRhoh-1	ACCCGTATTCTCGTACACCG
sgRhoh-2	ACCCACGGTGTACGAGAATA
sgRhoh-3	CCCTTCTATGGCATTGATGC

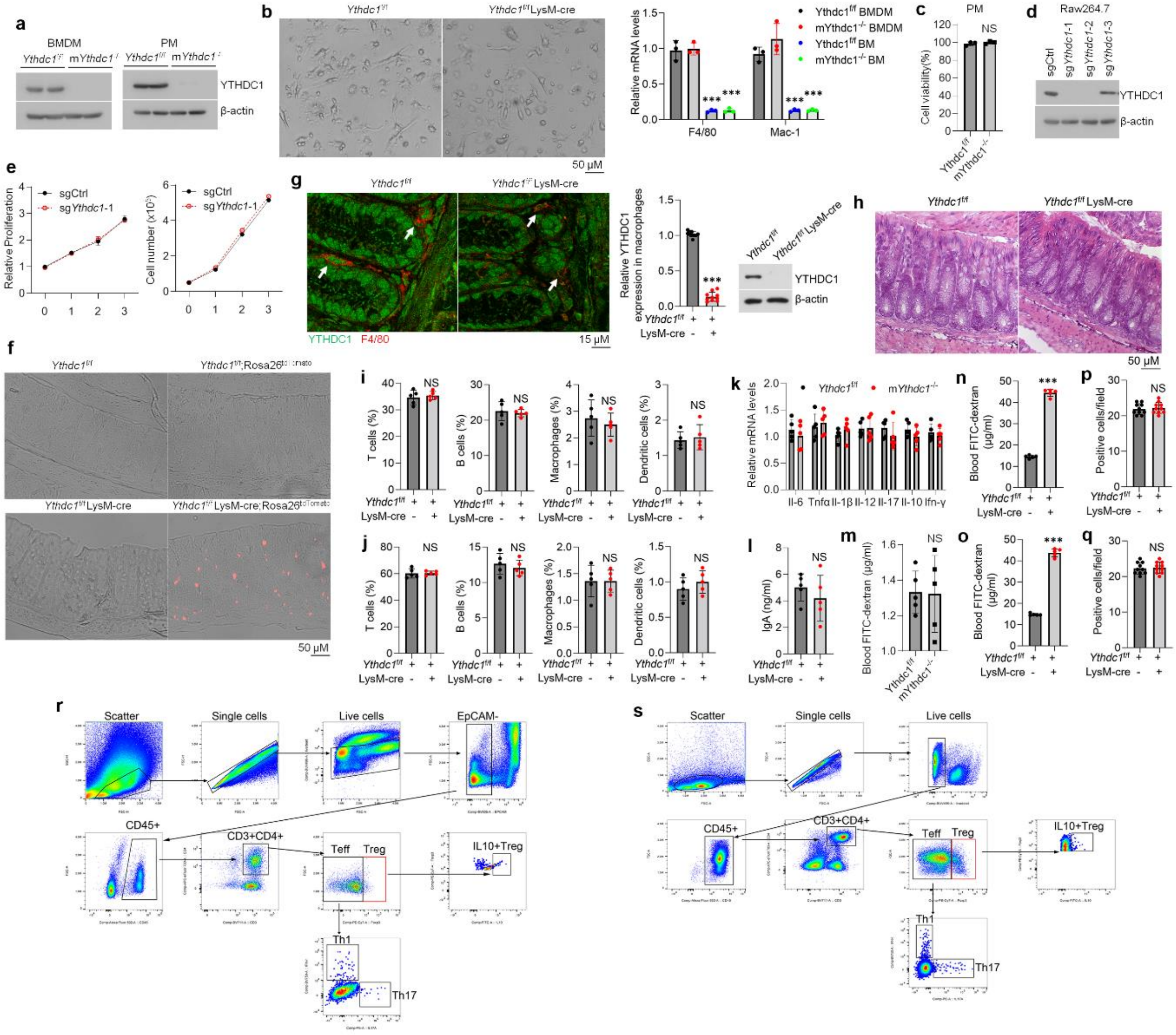

Supplemental figure 1. YTHDC1 deletion has no effects on macrophage development and colon tissues. (a) Western blot showing YTHDC1 protein levels in BMDMs (left) or PMs (right) derived from *Ythdc1*$^{f/f}$ and m*Ythdc1*$^{-/-}$ mice. Data are representative of three independent biological replicates. (b) Bright field of BMDMs (left) and qPCR analysis of *F4/80* and *Mac-1* levels in BMDMs and BMs derived from *Ythdc1*$^{f/f}$ and m*Ythdc1*$^{-/-}$ mice (right). (c) Cell viability of PMs isolated from *Ythdc1*$^{f/f}$ and m*Ythdc1*$^{-/-}$ mice after 24-hour culture. n = 3 for each group. (d-e) The knockout efficiency of sg*Ythdc1*-1-3 in Raw264.7 cells detected by western blot (d), and cell proliferation and numbers of sg*Ythdc1*-treated Raw264.7 on different days as indicated (e). n = 3 for each group. (f) Bright field and fluorescence images showing the Rosa^{26tdTomato} expression under macrophage-specific *Ythdc1* knockout conditions. (g) Immunofluorescence staining displaying YTHDC1 and F4/80 expression in the

mucosae of *Ythdc1*$^{f/f}$ and m*Ythdc1*$^{-/-}$ mice (left), and quantitative analysis of immunofluorescence staining (middle) and western blot analysis (right) of YTHDC1 expression in macrophages. (h) Histological examination of colon tissues from *Ythdc1*$^{f/f}$ and m*Ythdc1*$^{-/-}$ mice. (i-j) Immune cell composition in gut lamina propria (i) and gut-draining lymph node (j) derived from *Ythdc1*$^{f/f}$ and m*Ythdc1*$^{-/-}$ mice. (k-l) Cytokine (k) and IgA (l) levels of colonic mucosae detected by qPCR and Elisa in *Ythdc1*$^{f/f}$ and m*Ythdc1*$^{-/-}$ mice. (m) Epithelial barrier permeability detected by FITC-dextran assays in *Ythdc1*$^{f/f}$ and m*Ythdc1*$^{-/-}$ mice. (n-o) Epithelial barrier permeability detected by FITC-dextran assays in *Ythdc1*$^{f/f}$ and m*Ythdc1*$^{-/-}$ mice with DSS (n) or TNBS (o) treatment on day 14 or day 7, respectively. (p-q) Quantitative analysis of F4/80^{+} cells in *Ythdc1*$^{f/f}$ and m*Ythdc1*$^{-/-}$ mice with DSS (p) or TNBS (q) treatment, Related to figure 1f and 1m. (r-s) Flow cytometry gating strategies of gut lamina propria (r) and draining lymph node (s) for FACS analysis. n = 5 for each group. BMDM, bone marrow-derived macrophage; BM, bone marrow; PM, peritoneal macrophage; Ctrl, control. Data depict mean ± SD. Two-tailed students' *t*-test, one-way ANOVA and two-way ANOVA were performed for statistical analyses.

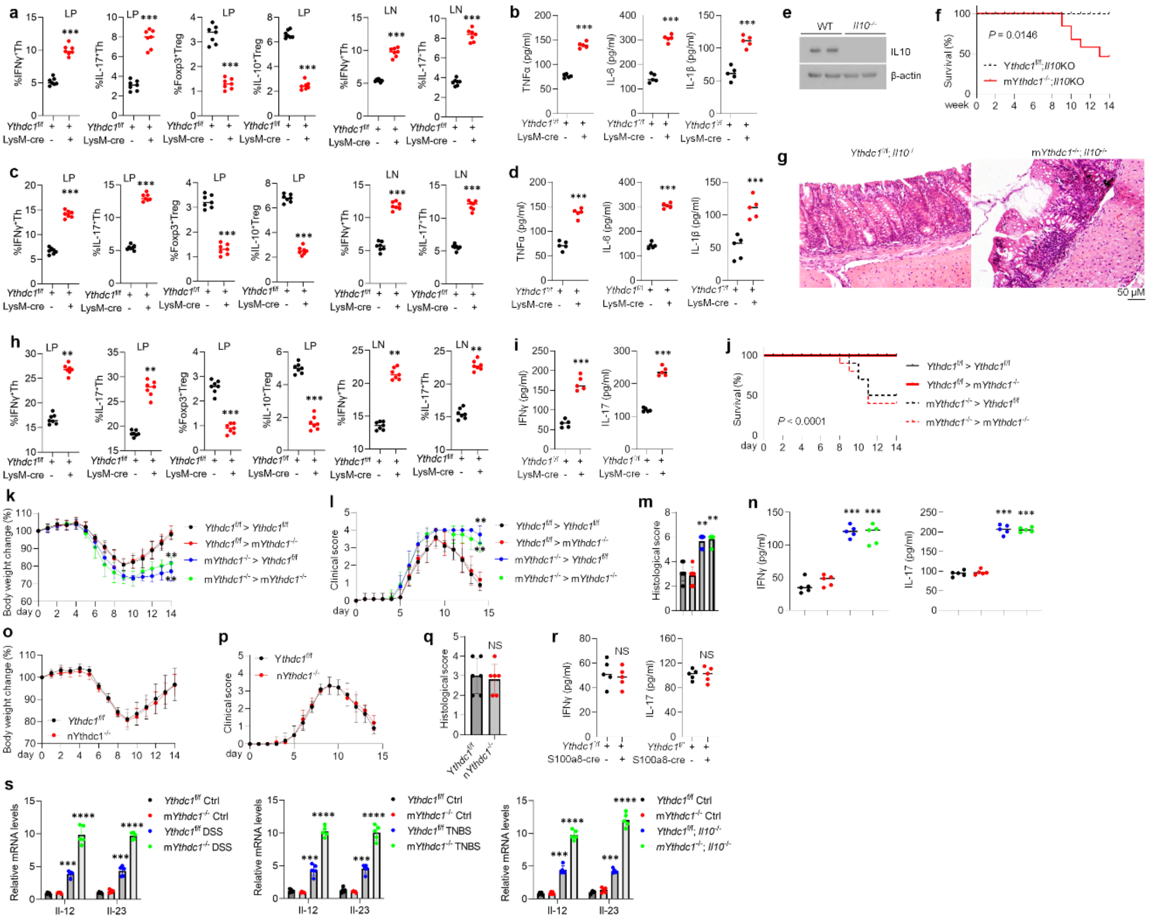

Supplemental figure 2. The roles of YTHDC1 in macrophages under colitis circumstances. (a) Cell frequencies of Th1, Th17 and Treg cells in gut lamina propria or lymph node from *Ythdc1*$^{f/f}$ and m*Ythdc1*$^{-/-}$ mice on day 9 post DSS treatment. n = 7 for each group. (b) TNFα, IL-6 and IL-1β levels tested by Elisa in the mucosae of *Ythdc1*$^{f/f}$ and m*Ythdc1*$^{-/-}$ mice on day 9 post DSS administration. n = 5 for each group. (c) Cell frequencies of Th1, Th17 and Treg cells in gut lamina propria or lymph node from *Ythdc1*$^{f/f}$ and m*Ythdc1*$^{-/-}$ mice on day 3 post TNBS treatment. n = 7 for each group. (d) TNFα, IL-6 and IL-1β levels tested by Elisa in the mucosae of *Ythdc1*$^{f/f}$ and m*Ythdc1*$^{-/-}$ mice on day 3 post TNBS administration. n = 5 for each group. (e) Western blot showing the protein levels of IL-10 in the colon mucosae of WT and *Il10*$^{-/-}$ mice. Data are representative of three independent biological replicates. (f) Survival curve of *Ythdc1*$^{f/f}$ and m*Ythdc1*$^{-/-}$ mice crossed with *Il10*$^{-/-}$ mice. n = 10 for each group. (g) Histological examination of *Ythdc1*$^{f/f}$ and m*Ythdc1*$^{-/-}$ mice crossed with *Il10*$^{-/-}$ mice at the age of 8 weeks. (h) Cell frequencies of Th1, Th17 and Treg cells in gut lamina

propria or lymph node from *Ythdc1*$^{f/f}$ and m*Ythdc1*$^{-/-}$ mice crossed with *Il10*$^{-/-}$ mice at the age of 8 weeks. n = 7 for each group. (i) IFNγ and IL-17 levels tested by Elisa in the mucosae of *Ythdc1*$^{f/f}$ and m*Ythdc1*$^{-/-}$ mice crossed with *Il10*$^{-/-}$ mice at the age of 8 weeks. n = 5 for each group. (j-l) Survival rate (j), body weight change (k) and clinical score (l) of bone marrow transplanted mice in the DSS-induced colitis model. n = 10 for each group. (m) Histological score assessment of bone marrow transplanted mice on day 9 post DSS administration. n = 6 for each group. (n) IFNγ and IL-17 levels tested by Elisa in the mucosae of bone marrow transplanted mice on day 9 post DSS administration. n = 5 for each group. (o-p) Body weight change (o) and clinical score (p) of *Ythdc1*$^{f/f}$ and n*Ythdc1*$^{-/-}$ mice in the DSS-induced colitis model. n = 10 for each group. (q) Histological score assessment of *Ythdc1*$^{f/f}$ and n*Ythdc1*$^{-/-}$ mice on day 9 post DSS administration. n = 6 for each group. (r) IFNγ and IL-17 levels tested by Elisa in the mucosae of *Ythdc1*$^{f/f}$ and n*Ythdc1*$^{-/-}$ mice on day 9 post DSS administration. (s) *Il-12* and *Il-23* levels in the gut macrophages from *Ythdc1*$^{f/f}$ and m*Ythdc1*$^{-/-}$ mice with DSS or TNBS treatment, or *Ythdc1*$^{f/f}$ and m*Ythdc1*$^{-/-}$ mice crossed with *Il10*$^{-/-}$ mice. n = 5 for each group. WT, wild type; KO, knockout; LP, lamina propria; LN, lymph node; Ctrl, control; TNBS, 2,4,6-trinitrobenzene sulfonic acid; DSS, dextran sulfate sodium. ** $p < 0.01$, *** $p < 0.001$ versus corresponding control group. Data depict mean ± SD. The log-rank test, two-tailed students' *t*-test, one-way ANOVA and two-way ANOVA were performed for statistical analyses.

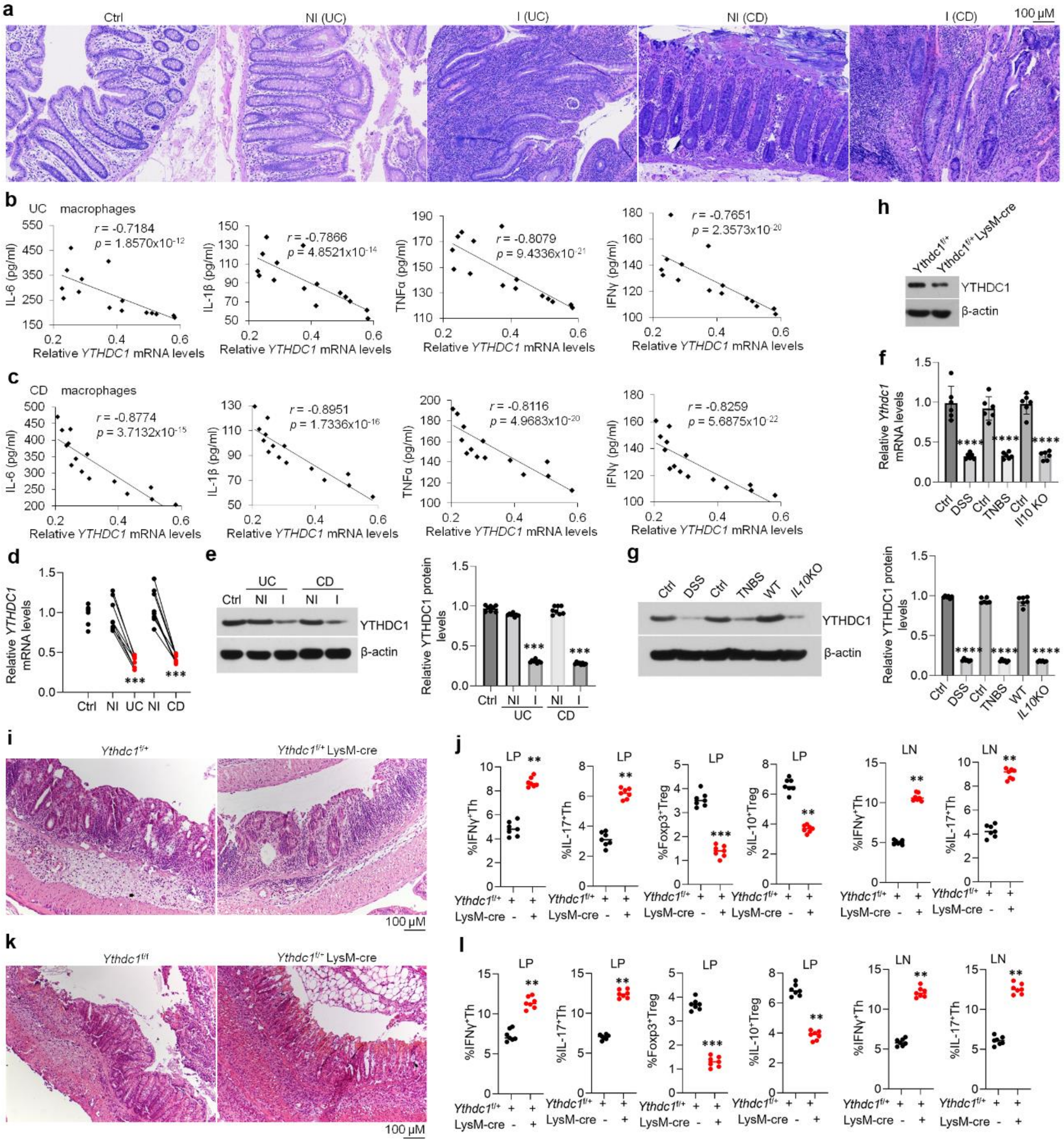

Supplemental figure 3. YTHDC1 deficiency in gut macrophages increases IBD severity. (a) HE staining of human colonic samples. (b-c) Correlation analyses of cytokines and *YTHDC1* levels in gut macrophages derived from UC (b) or CD (c) patients. n = 15 for each group. (d-e) Levels of YTHDC1 in FACS-purified macrophages from healthy individuals and IBD patients were tested by qPCR (d) and western blot (e). n = 8 for each group. (f-g) Levels of YTHDC1 in FACS-purified macrophages from control and colitis mice were tested by qPCR (f) and western blot (g). n = 6 for each group. (h) Western blot detection of YTHDC1 expression in BMDMs from *Ythdc1*$^{f/+}$ and m*Ythdc1*$^{+/-}$ mice. Data are representative of three independent biological replicates. (i) Histological examination of *Ythdc1*$^{f/+}$ and

m*Ythdc1*$^{+/-}$ mice on day 9 post DSS treatment. (j) Cell frequencies of Th1, Th17 and Treg cells in gut lamina propria or lymph node from *Ythdc1*$^{f/+}$ and m*Ythdc1*$^{+/-}$ mice on day 9 post DSS treatment. n = 7 for each group. (k) Histological examination of *Ythdc1*$^{f/+}$ and m*Ythdc1*$^{+/-}$ mice on day 3 post TNBS treatment. (l) Cell frequencies of Th1, Th17 and Treg cells in gut lamina propria or lymph node from *Ythdc1*$^{f/+}$ and m*Ythdc1*$^{+/-}$ mice on day 3 post TNBS treatment. n = 7 for each group. Ctrl, control; NI, non-inflammation; I, inflammation; UC, ulcerative colitis; CD, Crohn disease; LP, lamina propria; LN, lymph node. ** $p < 0.01$, *** $p < 0.001$, **** $p < 0.0001$ versus corresponding control group. Data depict mean ± SD. Two-tailed students' *t*-test and one-way ANOVA were performed for statistical analyses.

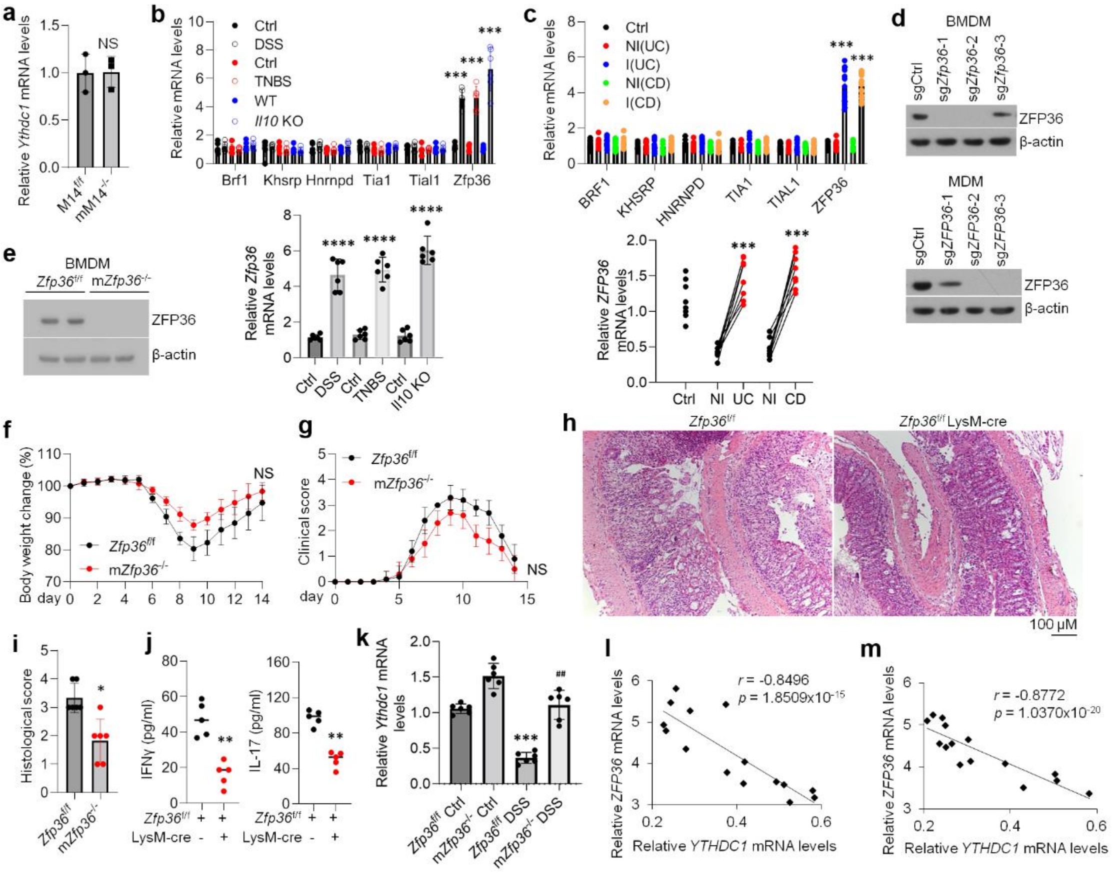

Supplemental figure 4. Functions of ZFP36 in macrophages. (a) *Ythdc1* mRNA levels detected by qPCR in macrophages with or without *Mettl14* depletion. n = 3 for each group. (b-c) Quantitative PCR showing mRNA binding protein expression in MACS-enriched gut macrophages (up) or FACS-enriched gut macrophages (bottom) from mice (b) or humans (c). n = 6 for mice groups and n = 15 for human groups. (d) Western blot showing ZFP36 expression in BMDMs (up) or MDMs (bottom) with sg*Zfp36*/*ZFP36* infection. Data are representative of three independent biological replicates. (e) Western blot showing ZFP36 expression in BMDMs with or without *ZFP36* deletion. Data represent three independent biological replicates. (f-g) Body weight change (f) and clinical score (g) of *Zfp36*$^{f/f}$ and m*Zfp36*$^{-/-}$ mice in the DSS-induced colitis model. n = 10 for each group. (h-i) HE staining (h) and histological score (i) of *Zfp36*$^{f/f}$ and m*Zfp36*$^{-/-}$ mice on day 9 after DSS treatment. n = 6 for each group. (j) IFNγ and IL-17 levels tested by Elisa in the mucosae of *Zfp36*$^{f/f}$ and m*Zfp36*$^{-/-}$ mice on day 9 after DSS treatment. (k) *Ythdc1* mRNA levels in gut macrophages from *Zfp36*$^{f/f}$ and m*Zfp36*$^{-/-}$ mice with or without DSS treatment. n = 5 for each group. (l-m) Correlation analyses of *ZFP36* and *YTHDC1* levels in gut

macrophages derived from UC (l) or CD (m) patients. n = 15 for each group. Ctrl, control; WT, wild type; NI, non-inflammation; I, inflammation; UC, ulcerative colitis; CD, Crohn disease; TNBS, 2,4,6-trinitrobenzene sulfonic acid; DSS, dextran sulfate sodium; BMDM, bone marrow-derived macrophage; MDM, monocyte-derived macrophage. ** $p < 0.01$, *** $p < 0.001$, **** $p < 0.0001$ versus corresponding control group. ## $p < 0.01$ versus corresponding DSS group. Data depict mean ± SD. Two-tailed students' *t*-test, one-way ANOVA and two-way ANOVA were performed for statistical analyses.

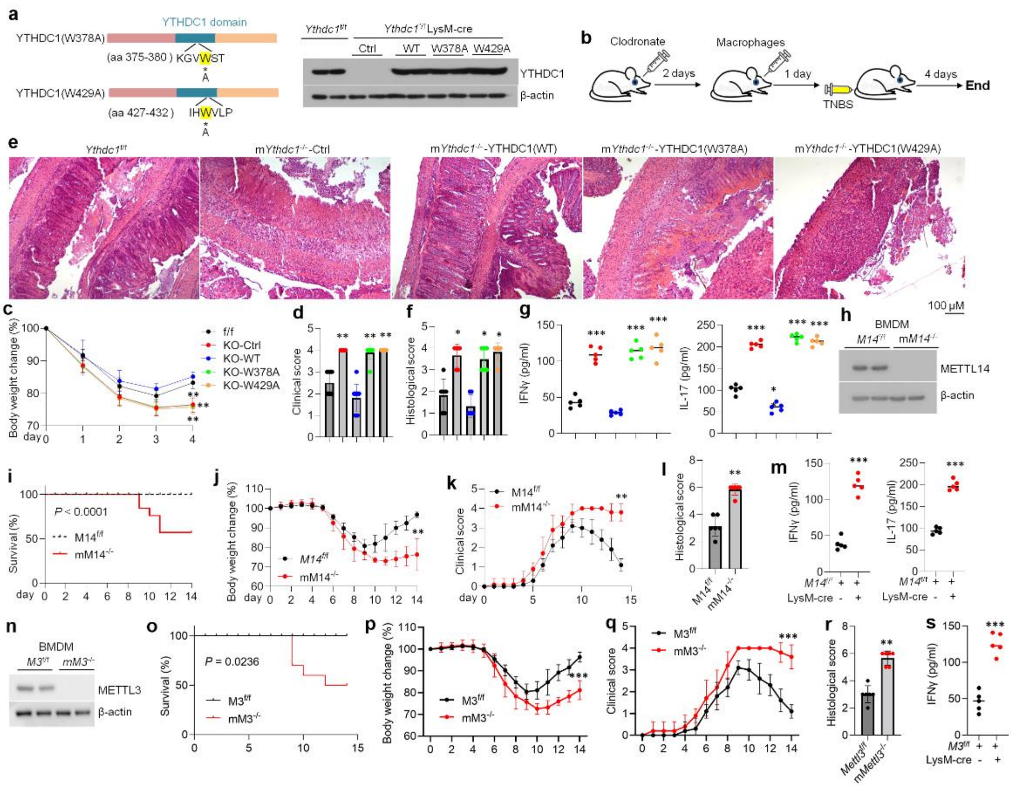

Supplemental figure 5. YTHDC1 exerts functional actions in a m^{6}A-dependent way. (a) Schematic of two YTHDC1 m^{6}A-binding site mutations resided in the YTH domain (left) and western blot detection of YTHDC1 expression in BMDMs with or without lentivirus infection as indicated (right). Data are representative of three independent biological replicates. (b) Schematic of macrophage depletion and reconstitution system followed by TNBS administration. (c-g) Wild type mice were injected with clodronate to delete endogenous macrophages and were reconstituted by *Ythdc1*$^{f/f}$ macrophages or *Ythdc1*$^{-/-}$ macrophages transduced with control-, WT *Ythdc1*-, W378A mutant-, or W429 mutant-lentivirus prior to TNBS treatment. These assays were performed: Body weight change (c) and clinical score (d), n = 10 for each group; HE staining (e) and histological score (f) on day 3 after TNBS treatment, n = 6 for each group; (g) IFNγ and IL-17 levels tested by Elisa in the colon mucosae on day 3 after TNBS treatment. n = 5 for each group. (h) METTL14 levels in M14$^{f/f}$ and M14$^{-/-}$ macrophages detected by western blot. Data are representative of three independent biological replicates. (i-k) Survival rate (i), body weight change (j) and clinical score (k) of M14$^{f/f}$ and mM14$^{-/-}$ mice in the DSS-induced colitis model. n = 10 for each group. (l) Histological score of M14$^{f/f}$ and mM14$^{-/-}$ mice on day 9 post DSS

administration. n = 6 for each group. (m) IFNγ and IL-17 levels tested by Elisa in the mucosae of $M14^{f/f}$ and $mM14^{-/-}$ mice on day 9 post DSS administration. (n) METTL3 levels in $M3^{f/f}$ and $M3^{-/-}$ macrophages detected by western blot. Data are representative of three independent biological replicates. (o-q) Survival rate (o), body weight change (p) and clinical score (q) of $M3^{f/f}$ and $mM3^{-/-}$ mice in the DSS-induced colitis model. n = 10 for each group. (r) Histological score of $M3^{f/f}$ and $mM3^{-/-}$ mice on day 9 post DSS administration. n = 6 for each group. (s) IFNγ and IL-17 levels tested by Elisa in the mucosae of $M3^{f/f}$ and $mM3^{-/-}$ mice on day 9 post DSS administration. n = 5 for each group. aa, amino acids; WT, wild type; Ctrl, control; KO, knockout; TNBS, 2,4,6-trinitrobenzene sulfonic acid; BMDM, bone marrow-derived macrophage; M14, Mettl14; M3, Mettl3. * $p < 0.05$, ** $p < 0.01$, *** $p < 0.001$ versus corresponding control group. Data depict mean ± SD. The log-rank test, two-tailed students' *t*-test, one-way ANOVA and two-way ANOVA were performed for statistical analyses.

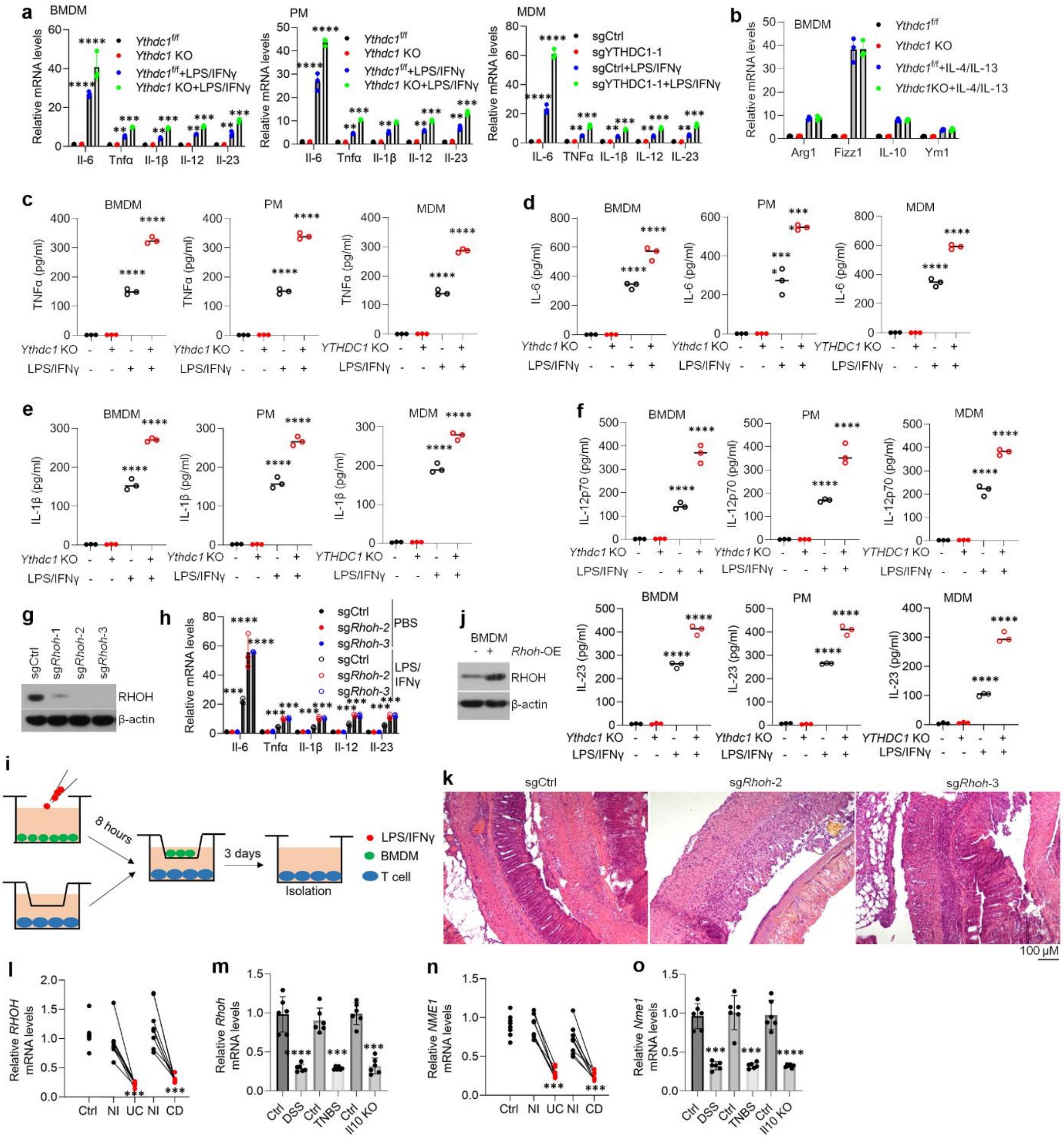

Supplemental figure 6. The roles of RHOH in macrophages and IBD. (a) M1 macrophage-related cytokines levels in *Ythdc1*$^{f/f}$ and *Ythdc1*$^{-/-}$macrophages with or without 8-hour LPS/IFNγ treatment. (b) M2 macrophage-related cytokines levels in *Ythdc1*$^{f/f}$ and *Ythdc1*$^{-/-}$macrophages with or without 8-hour IL-4/IL-13 treatment. (c-f) Elisa showing TNFα (c), IL-6 (d), IL-1β (e) or IL-12p70 (up) and IL-23 (down) (f) levels in *Ythdc1*$^{f/f}$ and *Ythdc1*$^{-/-}$macrophages with or without 8-hour LPS/IFNγ treatment. (g-h) Western blot detection of RHOH expression (g) and qPCR test of M1 macrophage-related cytokines levels (h) in sgCtrl- or sg*Rhoh*-lentivirus-infected

BMDMs with or without 8-hour LPS/IFNγ treatment. (i) Schematic of T cell and macrophage co-culture system with LPS/IFNγ treatment. (j) Western blot evaluation of RHOH in BMDMs with or without *Rhoh* overexpression. (k) HE staining of endogenous macrophage-depleted mice reconstituted with sgCtrl or sg*Rhoh*-lentivirus-infected macrophages on day 3 post TNBS administration. n = 3 for each group. (l-m) Quantitative PCR showing *RHOH*/*Rhoh* mRNA levels in FACS-enriched gut macrophages derived from IBD patients (l) or colitis mice (m). n = 8 for each human group, n = 6 for each mouse group. (n-o) Quantitative PCR showing *NME1*/*Nme1* mRNA levels in FACS-enriched gut macrophages derived from IBD patients (n) or colitis mice (o). n = 8 for each human group, n = 6 for each mouse group. BMDM, bone marrow-derived macrophage; PM, peritoneal macrophages; MDM, monocyte-derived macrophage; KO, knockout; OE, overexpression. *** $p < 0.001$, **** $p < 0.0001$ versus corresponding control group. Data depict mean ± SD. Two-tailed students' *t*-test, one-way ANOVA and two-way ANOVA were performed for statistical analyses.

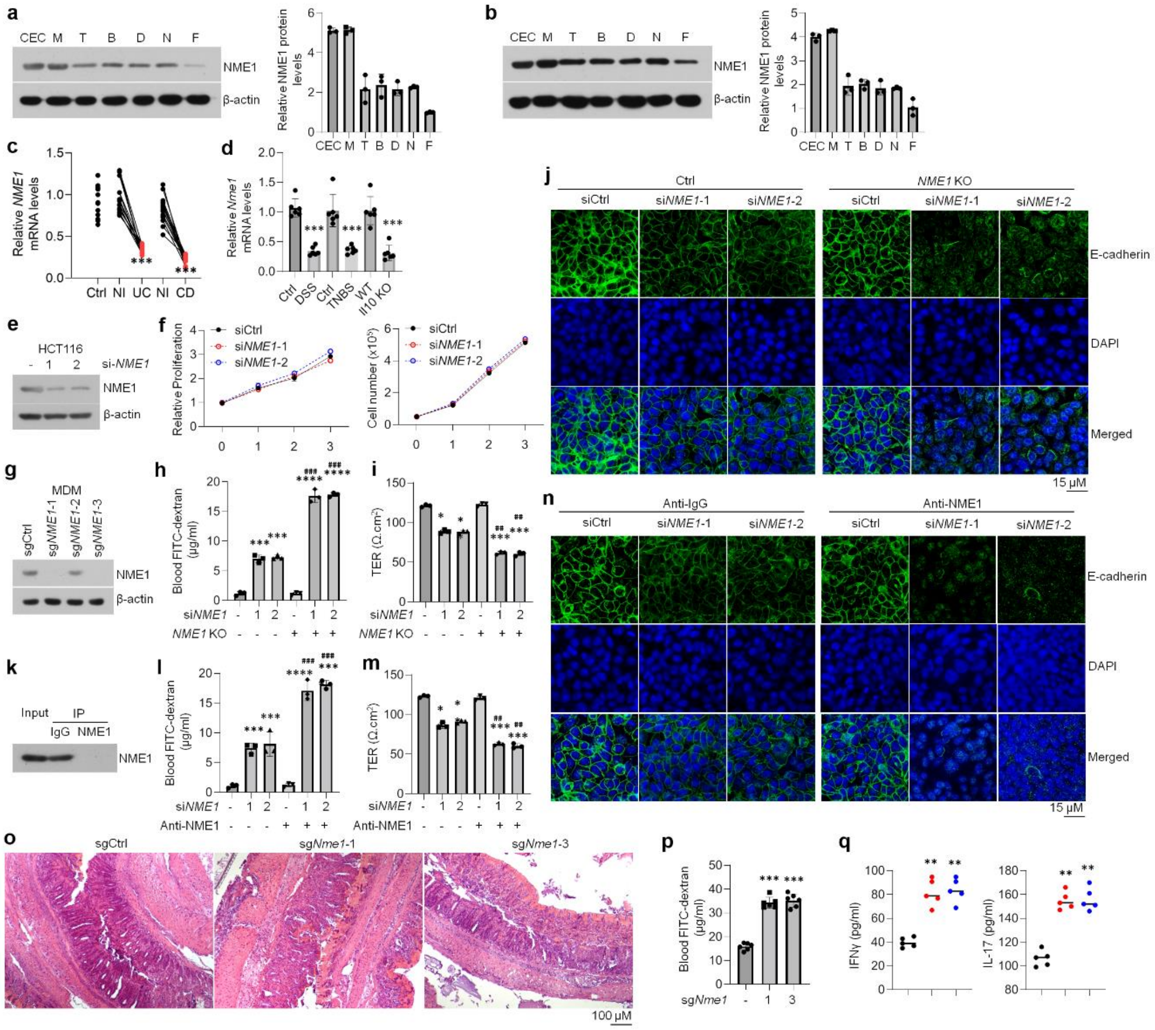

Supplemental figure 7. The roles of NME1 in macrophages and IBD. (a-b) NME1 protein levels in colonic epithelial cells, macrophages, T cells, B cells, dendritic cells, neutrophils or fibroblasts from human (a) and mouse (b) detected by western blot. n = 3 for each group. (c-d) *NME1*/*Nme1* levels in colonic epithelial cells derived from human (c) or mouse (d) assessed by qPCR. n = 15 for each human group, n = 6 for each mouse group. (e-f) Western blot detection of NME1 levels (e) and cell proliferation and cell number assessments (f) in HCT116 cells transfected with or without si*NME1*. n = 3 for each group. (g) Western blot showing NME1 expression in MDMs with or without sg*NME1*-lentivirus infection. n = 3 for each group. (h-j) FITC-dextran permeability assays (h), trans-epithelial electrical resistance measurements (i) or immunofluorescence staining (j) of siRNAs-transfected HCT116 cells co-cultured with or without *NME1*$^{-/-}$MDMs. n = 3 for each group. (k) Western

blot showing NME1 expression in the macrophage culture medium with IgG or NME1 antibodies treatment. For immunodepletion of NME1 in medium, NME1 was immunoprecipitated using anti-NME1 antibodies supplemented with protein A/G magnetic beads. n = 3 for each group. (l-n) FITC-dextran permeability assays (l), trans-epithelial electrical resistance measurements (m) or immunofluorescence staining (n) of siRNAs-transfected HCT116 cells co-cultured with or without anti-NME1 antibody-treated medium from MDMs. n = 3 for each group. (o-q) Wild type mice were injected with clodronate to delete endogenous macrophages and reconstituted by macrophages infected with sgCtrl- or sg*Nme1*-lentivirus prior to TNBS treatment, related to Figure 5p-r. These assays were performed: HE staining of mice on day 3 post TNBS treatment (o); Blood FITC-dextran detection from mice on day 3 post TNBS treatment (p), n = 6 for each group; IFNγ and IL-17 levels tested by Elisa in the mucosae of mice, n = 5 for each group. CEC, colonic epithelial cell; M, macrophage; T, T cell; B, B cell; D, dendritic cell; N, neutrophil; F, fibroblast; Ctrl, control; WT, wild type; NI, non-inflammation; I, inflammation; UC, ulcerative colitis; CD, Crohn disease; TNBS, 2,4,6-trinitrobenzene sulfonic acid; DSS, dextran sulfate sodium; MDM, monocyte-derived macrophage; KO, knockout. * $p < 0.05$, ** $p < 0.01$, *** $p < 0.001$, **** $p < 0.0001$ versus corresponding control group; ## $p < 0.01$, ### $p < 0.001$ versus si*NME1* alone group. Data depict mean ± SD. Two-tailed students' *t*-test, one-way ANOVA and two-way ANOVA were performed for statistical analyses.

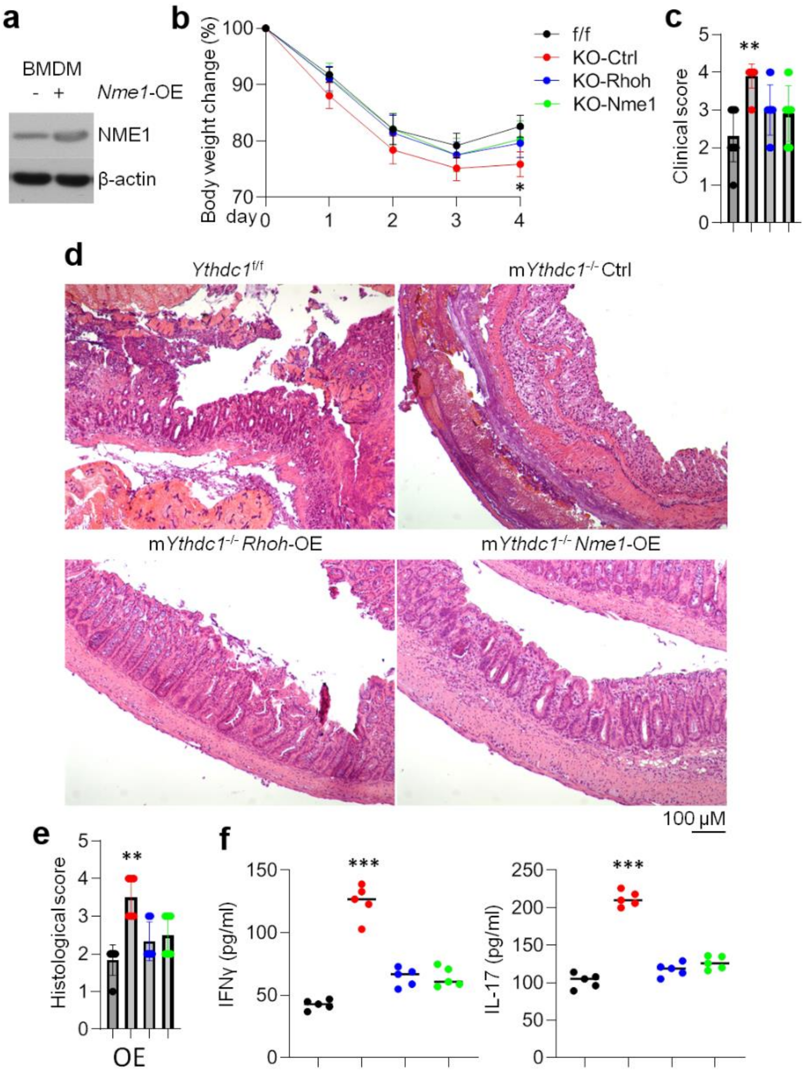

Supplemental figure 8. Overexpression of *Rhoh* or *Nme1* in macrophages ameliorates colitis severity. (a) Western blot showing NME1 levels in BMDMs with or without *Nme1* overexpression. (b-f) Wild type mice were injected with clodronate to delete endogenous macrophages and reconstituted by *Ythdc1*$^{f/f}$ macrophages or *Ythdc1*$^{-/-}$ macrophages transduced with control-, *Rhoh*- or *Nme1*-lentivirus prior to TNBS treatment. These assays were performed: Body weight change (b) and clinical score on day 3 post treatment (c), n = 10 for each group; HE staining (d) and histological score (e) on day 3 after TNBS treatment, n = 6 for each group; (f) IFNγ and IL-17 levels tested by Elisa in the colon mucosae on day 3 after TNBS treatment. n = 5 for each group. BMDM, bone marrow-derived macrophage; OE, overexpression; Ctrl, control. ** $p < 0.01$, *** $p < 0.001$ versus corresponding control group. Data depict mean ± SD. One-way ANOVA and two-way ANOVA were performed for statistical

analyses.

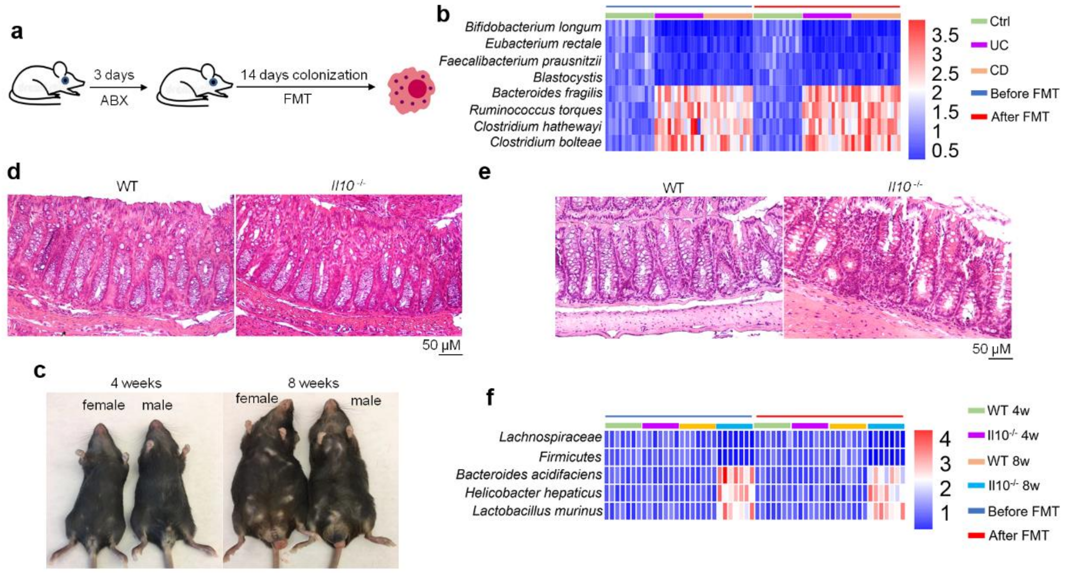

Supplemental figure 9. Fecal microbiota transplantation (FMT). (a) Schematic of fecal microbiota transplantation workflow in wild type mice. (b) Wild type mice were transplanted with microbiotas from healthy volunteers or IBD patients. Fecal samples were collected before FMT from human or after FMT from transplanted mice. Relative abundances of bacteria species were detected by qPCR. n = 15 for each group. (c) Images of 4-week-old and 8-week-old *Il10*$^{-/-}$ mice. (d-e) HE staining of colon tissues from 4-week-old (d) and 8-week-old (e) wild type or *Il10*$^{-/-}$ mice. (f) Wild type mice were transplanted with microbiotas from 4-week-old or 8-week-old wild type/*Il10*$^{-/-}$ mice. Fecal samples were collected before FMT from donor mice or after FMT from transplanted mice. Relative abundances of bacteria species were detected by qPCR. n = 7 for each group.

Methods and materials

Animals

Ythdc1$^{flox/flox}$ mice possessing *loxP* sites flanking exons 5-9 of the *Ythdc1* gene, LysM-Cre transgenic mice, S100a8-Cre transgenic mice, *Il10* knockout mice and Rosa^{26tdTomato} mice in a C57BL/6 background were all purchased from Jackson Laboratory. *Zfp36*$^{flox/flox}$ mice bearing *loxP* sites between exon 2 in the *Zfp36* gene were provided by Perry J. Blackshear at National Institutes of Health[1]. *Mettl14*$^{flox/flox}$ and *Mettl3*$^{flox/flox}$ mice harboring *loxP* sites were from Shanghai Model Organisms Center. 6 to 8-week old mice were used for animal experiments. All mice were housed in the SPF facilities with a 12 light–12 dark cycle in animal room. Littermate controls were used for all studies. Both female and male mice were involved together, since the preliminary data showed that gender is not a confounding contributor in colitis development. All animal investigations were approved by the Institutional Animal Care and Use Committee of Shanxi Medical University (#2021GLL054).

Cell culture

L929 (CCL-1), HEK293T (CRL-3216), RAW 264.7 (TIB-71), HCT116 (CCL-247), CMT93 (CCL-22) and primary human fibroblast (PCS-201-012) cell lines were purchased from ATCC. RAW 264.7, HEK293T, HCT116, CMT93 and L929 cells were cultured in DMEM medium supplemented with 10% FBS and 1% penicillin/streptomycin (P/S). Primary human fibroblasts were plated with fibroblasts basal medium including 10% FBS and 1% P/S. All cells were cultured under 37 °C and 5% CO_2 conditions. Macrophages were treated with LPS (100 ng/ml)/IFNγ (100 ng/ml) or IL-4 (100 ng/ml)/IL-13 (100 ng/ml) at different time points as described. In parallel experiments, nuclear and cytoplasmic fractions were separated and extracted using the NE-PER™ Nuclear and Cytoplasmic Extraction Reagents (Thermo Fisher Scientific).

Human monocyte-derived macrophages (MDMs).

MDMs were cultured as described before[2]. In brief, mononuclear cells in human blood samples were purified using Ficoll-Paque density gradient cell

separation (GE Healthcare) following the manufacturer’s protocol. Pan Monocyte Isolation Kit (Miltenyi Biotech) was used to further enrich monocytes from blood mononuclear cells. The purified monocytes were grown in RPMI 1640 supplemented with 1% P/S, 10% FBS, 1% sodium pyruvate, 1% non-essential amino acids, glutamine (2 mM), and human recombinant M-CSF (20 ng/ml, PeproTech). Monocytes would differentiate into macrophages in 7 days.

RNA interference and Transfections.

HCT116 cells were transfected with siRNAs targeting *NME1* using Lipofectamin 3000 (Invitrogen) for 36 hours. siRNA sequences of *NME1* are 5’-GGCUGUAGGAAAUCUAGUU-3’ and 5’-GGAUUCCGCCUUGUUGGUC-3’. The control siRNA sequence is 5’-GGCUGUAGAAGCUAUAGUU-3’.

Colitis models

Colitis models were established according to previous publications[3]. For 2,4,6-trinitrobenzene sulfonic acid (TNBS)-induced colitis model, mice were treated with TNBS solution (1%) which was applied on the back skin for pre-sensitization. 8 days later, these presensitized mice were fasted overnight and administrated with TNBS (100 mg/kg) dissolved in 50% alcohol through intrarectal injection under anesthesia, using a 1 ml syringe with an 18-gauge straight gavage needle. 50% alcohol was injected into control mice. Mice were positioned head down for 1 min after TNBS injection. For dextran sulfate sodium (DSS) model establishment, mice were provided 2.5% DSS (Thermo Fisher Scientific) dissolved in autoclaved tap water for 7 days followed by a 7-day tap water treatment based on published procedure[3]. Survival, body weights, rectal bleeding and stool consistency were monitored every day. Clinical and histological scores were evaluated as described before[3]. Scoring system for clinical index based on stool consistency, weight loss and the severity of intestinal bleeding. *Il10* KO mice develop spontaneous colitis in 6-8 weeks after birth[4].

Bone marrow-derived macrophage (BMDM) and peritoneal macrophage (PM)

BMDMs were cultured and isolated following previous publications[5]. Briefly, bone marrow cells were harvested from the legs of mice by cold PBS, followed by red

blood cells elimination. Cells were cultured with 30% L929 conditioned media for differentiation. BMDMs were ready to use after being cultured for 7 days. In other experiments, isolated bone marrow cells were infected with a lentivirus for 48 hours before differentiation.

For peritoneal macrophage isolation, mice were intraperitoneally injected with 5 ml cold PBS followed by PBS fluids collection. Cell suspension was centrifuged to pellet macrophages. Peritoneal macrophages were cultured in RPMI 1640 media with 10% heat-inactivated FBS and 1% P/S.

Histology and Immunostaining

Fresh colon tissues were harvested, cut longitudinally, flushed with cold PBS and fixed immediately in 4% formaldehyde overnight. Then, the colon tissues were prepared as “Swiss rolls” followed by dehydration with ethanol, vitrification with xylene and embedding with paraffin. The tissue blocks were then cut into 4 µm sections. For H&E staining, slides were treated with hematoxylin and eosin respectively. For immunostaining, slides were retrieved with citric acid buffer and then incubated with primary antibodies and fluorescence-conjugated secondary antibodies. For cultured macrophages and HCT116 cell line, cells were fixed by 4% formaldehyde for 15 min at room temperature and then permeabilized by 0.2% NP-40 buffer prior to antibody incubation. Nucleuses were stained with DAPI. The tissues were imaged under a microscope.

Bone marrow transplantation

Bone marrow transplantation was performed according to published procedure[6]. Briefly, recipient mice were subject to γ-irradiation of 1050 rads (200 rads/min) to deplete original bone marrow cells. 6 hours after irradiation, the recipient mice were transplanted with bone marrow cells ($5x10^6$ cells/mouse) from donor mice through retra-orbital injection. Two months later, the transplanted mice were ready for colitis induction.

Fecal microbiota transplantation (FMT)

FMT was performed based on a published paper[7]. Briefly, donor feces from IBD patients and healthy individuals were harvested and dissolved in anaerobic PBS

containing 10% glycerol under anaerobic conditions. To eliminate the original microbiota, wild type C57BL/6J mice were administered a cocktail of antibiotics (1 mg/ml ampicillin, 5 mg/ml streptomycin and 1 mg/ml colistin) dissolved in the autoclaved water. Freshly harvested fecal suspension was administered to recipient antibiotics-treated wild type mice by oral gavage (200 µl per mouse). Transplanted mice were subsequently housed in sterile cages with sterile chow and water. Two weeks later, these transplanted animals were ready for experiments.

Lentiviral and plasmid constructs.

Lentivirus overexpressing mouse YTHDC1, RHOH or NME1 and human RHOH were constructed by inserting the coding sequence of mouse *Ythdc1* [NM_177680.4], *Rhoh* [NM_001081105.2], *Nme1* [NM_008704.3] or human *RHOH* [NM_001278359.2] cDNA into pLV[Exp]-Neo-EF1A lentiviral vector (VectorBuilder). Lenti-vector and packaging plasmids were transfected into HEK293T cells together. After 48 hours, packaged lentivirus in culture medium of HEK293T cells was harvested and transduced into macrophages supplemented with polybrenes (4 µg/ml). Site-specific mutations of *Ythdc1* were carried out using a QuickChange Site-Directed Mutagenesis Kit (Agilent) according to the manufacturer's procedure. pRP-CMV vector and pRP-Zfp36 plasmid were provided kindly by Yanchun Li (The University of Chicago). For pGL3-*Rhoh* or pGL3-*Nme1* plasmid generation, the fragment possessing m^6A element in mouse *Rhoh* or *Nme1* cDNA was cloned to the downstream of Luc gene using pGL3-Promoter (Promega) vector. pGL3-*Rhoh*-Mut or pGL3-*Nme1*-Mut plasmids were constructed via mutating the sequence 5'GGACT3' to 5'GGTCT3' or 5'GAACT3' to 5'GATCT3' in pGL3-*Rhoh* or pGL3-*Nme1* vector by the Mutagenesis Kit, respectively. For mouse pGL3-*Ythdc1* or human pGL3-*YTHDC1* plasmid generation, the fragment harboring AU-rich element in the 3'UTR of mouse *Ythdc1* or human *Ythdc1* cDNA was subcloned to the downstream of Luc gene as well. pGL3-*Ythdc1*-Mut or pGL3-*YTHDC1*-Mut plasmids were generating by mutating the AU-rich motif sequence 5'UAUUUAUA3' to 5'UAAAAAUA3' or 5'AAAUUUAUUU3' to 5'AAAAAAAUUU3', respectively. All of the mutations were confirmed by DNA

sequencing. Primers were listed Table S2.

Luciferase reporter assay.

BMDMs plated in 12-well plates were co-transfected with 500 ng pRP-CMV, pRP-Zfp36, pGL3-Promoter, pGL3-*Ythdc1*-WT/pGL3-*YTHDC1*-WT or pGL3-*Ythdc1*-Mut/pGL3-*YTHDC1*-Mut plasmids via jetPEI-Macrophage DNA Transfection Reagent (Polyplus-transfection). In parallel experiments, BMDMs were co-transfected with 500 ng pRP-CMV, pRP-*Mettl14*, pGL3-Promoter, pGL3-*Rhoh*-WT/pGL3-*Nme1*-WT or pGL3-*Rhoh*-Mut/pGL3-*Nme1*-Mut plasmids. After 24 hours, BMDMs were harvested, and luciferase activities were assessed using a Luciferase Assay System kit (Promega) following the manufacture's protocols. pRL-TK renilla luciferase reporter vectors were transfected to serve as internal controls.

CRISPR/Cas9-modulated gene knockout.

sgRNA sequences targeting the *Ythdf1* gene were inserted into lentiCRISPRv2 vector (Addgene) by the BsmBI restriction enzyme. Lenti-vector and packaging plasmids were transfected into HEK293T cells together. After 48 hours, packaged lentivirus in culture medium of HEK293T cells was harvested and transduced into macrophages supplemented with polybrenes (4 μg/ml). Details on sgRNA were provided in Table S2.

Macrophage depletion and reconstitution.

Macrophages in mice were depleted using clodronate-containing liposomes following previous publications[5, 8]. In brief, mice were intravenously administered 5 mg/ml clodronate-liposomes (0.2 ml/mouse) from Encapsula NanoSciences. 48 hours later, mice were reconstituted with fresh $2x10^6$ BMDMs suspended evenly in PBS with or without lentivirus infection via intravenous injection. 24 hours after reconstitution, these mice were used for the induction of colitis using TNBS.

Colonic epithelial cells, lamina propria cells, macrophages, T cells, B cells, dendritic cells and neutrophils isolation

Fresh mice colon tissues were harvested, cut longitudinally, flushed with cold PBS and cut into 3 mm fragments. Freshly harvested human colon biopsies were cut

into 3 mm fragments on ice directly. Colonic epithelial cells were isolated as described before[9]. In brief, the fragmented tissues were rotated in cold PBS including EDTA (10 mM) for 30 min in cold room. After centrifugation and multiple washes with cold PBS, tissues were pipetted up and down to separate epithelial layer and lamina propria, followed by crypts enrichment through a cell strainer (70-μm). The enriched epithelial cells were then purified by 20%/40% Percoll. Colonic lamina propria cells were enriched as described[10]. Briefly, the fragmented tissues were rotated in PBS with DTT (1 mM) for 10 min, followed by shaking in PBS with HEPES (10 mM) and EDTA (30 mM). The tissues were then digested by collagenase VIII (Sigma-Aldrich) (150 U/ml). Cell suspensions were filtered by a 70 μm cell strainer, and then enriched by centrifugation using the 40%/80% Percoll.

Human macrophages in the colonic lamina propria were purified by CD14 (Miltenyi Biotec) and CD11B magnetic beads (Miltenyi Biotec). Mouse macrophages in the colonic lamina propria were purified by F4/80 (Miltenyi Biotec) and CD11b magnetic beads. Human T cells, B cells and dendritic cells were isolated from blood by T Cell Isolation Kit (Miltenyi Biotec), B cell isolation Kit (Miltenyi Biotec) and dendritic cell isolation kit (Miltenyi Biotec). Mouse T cells, B cells and dendritic cells were isolated from the spleen by T Cell Isolation Kit (Miltenyi Biotec), B cell isolation Kit (Miltenyi Biotec) and dendritic cell isolation kit (Miltenyi Biotec). Neutrophils from human or mouse blood were isolated by human neutrophil isolation kit (Miltenyi Biotec) or mouse neutrophil isolation kit (Miltenyi Biotec), respectively.

Flow cytometry and cell sorting

Flow cytometry analysis was carried out according to previous publications[11]. Briefly, samples were treated with anti-CD16/32 antibody (eBioscience), followed by cell surface marker staining. For intracellular staining, samples were fixed and permeabilized before PMA (50 ng/ml), ionomycin (500 ng/ml) and brefeldin A (2 μg/ml) treatments. The Live and Dead Violet Viability Kit (Invitrogen) was used to separate dead cells. Antibodies for flow cytometric analyses in mouse samples were: anti-CD45-Alexa Fluor 532 (clone 30-F11, Invitrogen), anti-CD11B-Brilliant Violet 711 (clone M1/70, Biolegend), anti-F4/80-PE (clone BM8, Biolegend),

anti-I-A/I-E-Alexa Fluor 700 (clone M5/114.15.2, Biolegend), anti-CD4-APC-eFluor 780 (clone GK1.5, Invitrogen), anti-FOXP3-PE-Cyanine7 (clone FJK-16s, Invitrogen), anti-IL-17A-PE (clone eBio17B7, eBioscience), anti-IFN-γ-BV750 (clone XMG1.2, BD), anti-CD3- BV711 (clone 17A2, Biolegend), anti-CD326-BV605 (clone G8.8, Biolegend), anti- IL-10-FITC (clone JES5-16E3, Biolegend). Antibodies for flow cytometric analyses in human samples were: anti-CD45-Brilliant Violet 510 (clone 2D1, Biolegend), anti-CD11B-Brilliant Violet 711 (clone M1/70, Biolegend), anti-CD14-PE (clone M5E2, Biolegend), anti-HLA-DR-Alexa Fluor 700 (clone L243, Biolegend). Cytek Aurora (Cytek Biosciences) and Bigfoot (Thermo Fisher Scientific) were used for cell detecting and sorting respectively. Data were analyzed by FlowJo software V10.8.1.

RNA affinity chromatography

RNA affinity chromatography experiments were implemented as previously described[12]. In brief, ssRNA oligonucleotides possessing adenosine or m^6A were labelled with biotin and synthesized from GE Dharmacon (Lafayette). The ssRNA baits were boiled for 10 min and then placed on ice instantly for denaturation. RNA oligonucleotide (0.4 pmol) was rotated in binding buffer with 50 μl streptavidin magnetic beads (Thermo Fisher Scientific) in cold room for 4 hours. 200 μg nuclear extract from macrophages was added into the RNA bait-bead mixtures at a final volume of 400 μl in binding buffer for another 12-hour incubation. After several washes, RNA–protein complexes were lysed in RIPA buffer and subjected to analysis by western blot.

Western blot

Cells or tissues were lysed in RIPA buffer (Invitrogen) with protease inhibitor cocktail (Roche). Lysates were heated for 10 min at 95 °C for denaturation, and quantification of protein was conducted by a PierceTM BCA Kit. Denatured protein was separated by 4-12% SDS-PAGE gels and then transferred to PVDF membranes (Millipore). Membranes containing protein were blocked by 5% milk buffer at room temperature for 1 hour prior to primary antibodies incubation at 4 °C overnight. On day 2, after washes with TBST buffer, membranes were incubated with

HRP-conjugated secondary antibodies at room temperature for 1 hour. Pierce ECL Substrate was used to detect protein bands. Signals were observed by films or an imaging system (Bio-Rad). Densitometric quantitation of bands were performed by the ImageJ software.

RT-qPCR and m^6A-IP-qPCR

Total RNAs from cells or tissues were isolated by TRIzol reagent (Invitrogen), followed by reverse transcription using a reagent kit (TOYOBO) to synthesize cDNA. Quantitative PCR (qPCR) was carried out using a SYBR real-time PCR kit (TOYOBO). Relative gene levels were normalized to GAPDH expression. For m^6A-IP-qPCR, enriched and fragmented mRNAs were incubated with anti-m^6A antibody and purified by the EpiMark N^6-Methyladenosine Enrichment Kit (NEB). 10% of fragmented mRNAs were saved for input samples. The enriched m^6A levels were normalized to the input controls. Relative amount of each gene was calculated by the $2^{-\Delta\Delta Ct}$ method. Primers for qPCR are presented in Table S2.

Cross-linking and RNA Immunoprecipitation-qPCR (CLIP-qPCR)

CLIP assays were performed according to previous publications[8] with slight modifications. Briefly, macrophages were stimulated with 4-thiouridine (100 mM) in 37°C for 14 hours, followed by cross-linking with UV irradiation at 0.15 J/cm^2 on ice. Cells were lysed in lysis buffer and then harvested for immunoprecipitation. Before immunoprecipitation, RNase T1 (0.1 U/μl) was added for 10-min incubation at 22°C, and 10% of lysates were saved as input controls, while the other 90% of samples were mixed with antibody-protein G magnetic bead complex for 1-hour rotation in cold room. Immunoprecipitated RNAs were extracted with TRIzol reagents and quantified by qPCR.

Assessment of transcription rate

Transcription rate experiments were implemented following a published paper[13]. Briefly, 5-Ethynyl Uridine (EU, 0.5 mM) was added to macrophages for 0-min, 20-min, 40-min or 60-min treatments separately. Total RNAs were isolated by TRIzol reagent and nascent RNAs were purified by the Click-iT Nascent RNA Capture Kit (Invitrogen) according to the manufacturer's instructions. RNA amounts

were evaluated by RT-qPCR.

mRNA decay assay

Macrophages were added with actinomycin D (5 mg/ml) for 0-hour, 2-hour, 4-hour or 8-hour treatments prior to total RNAs extraction. mRNA amounts of each time point were detected by RT-qPCR. The mRNA level of 0 hour was used for normalization.

Cell viability and proliferation assays

Cell viability was measured using the CellTiter-Glo 2.0 kit (Promega) following the standard protocols. Cell proliferation at different time points was detected by the Cell Proliferation Assay kit (Sigma) according to the manufacturer's instructions. The initial number of cells for each group is 5000 at day 0.

NF-κB activity assay.

NF-κB activity of macrophages was measured using NF-κB luciferase reporter kit (BPS Bioscience). Macrophages with or without LPS/IFNγ stimulations were co-transfected with NF-κB luciferase reporter as well as pRL-TK renilla luciferase reporter. The Dual Luciferase Assay System was used to monitor luciferase activities.

Permeability Measurement

Colonic epithelial cells and macrophages were co-cultured in 0.4 μm Transwell chambers (Corning) for permeability assays until monolayers of epithelial cells were formed. Transepithelial electrical resistance (TER) of colonic epithelial cell monolayer was measured using an electrical resistance system (Millicell ERS-2 Voltohmmeter, Millipore). In another experiment, 200 μL of 10 mg/ml 4000 Da FITC-Dextran (Sigma) was added to the apical side of cell monolayer. 4 hours later, 100 μL aliquots of culture medium were collected from the basal chamber for measurements[14]. For *in vivo* studies, mice were fasted for 6 hours prior to being gavaged with 4000 Da FITC-dextran orally at 200 mg/kg. 2 hours later, blood was harvested from each mouse for detections[14]. Serum or medium FITC-dextran was monitored by a fluorescence spectrometer at 530 nm wavelength.

Cytokines detection

TNFα, IFNγ, IL-6, IL-17, IL-1β, IL-12p70, IL-23 and IgA concentrations of cells,

tissues or cell culture media were measured by various ELISA kits (Biolegend) following the manufacturer's protocols. For cells and tissues, 100,000 cells or 1 mg tissues were lysed in 300 µl complete extraction buffer (150 mM NaCl, 100 mM Tris (pH 7.4), 1mM EDTA, 1 mM EGTA, 0.5% sodium deoxycholate, 1% Triton X-100 and protease inhibitor cocktail) and homogenized with a homogenizer on ice, followed by 2-hour rotation in cold room. Lysates were then centrifuged at 13,000 rpm in cold room for 20 min and supernatants were saved for further analysis.

Cell co-culture

Transwell inserts (Corning, Polycarbonate, 0.4 mm) were selected to establish the co-culture system. For HCT116 and macrophage co-culture, BMDMs were plated in the 24-well carrier plate at the density of $1x10^6$/well. HCT116 cells with or without si*NME1* transfection were cultured into the insert at the density of $1x10^5$/well until monolayers were formed, followed by TER measurement and FITC-dextran permeability assay. In another experiment, HCT116 cells were cultured in the plate and BMDMs were plated into the insert for immunofluorescence staining. For macrophage and T cell co-culture, naive $CD4^+$ T cells were enriched from the spleen of wild type mice using CD4 T cell isolation kit (Miltenyi Biotec) and cultured in the anti-CD3 and anti-CD28 coated plate. BMDMs were plated and treated with LPS/IFNγ for 8 hours, and then the supernatant of BMDMs was pipetted out and BMDMs were washed 3 times with cold PBS to remove extracellular LPS and cytokines. T cells were grown in the 24-well carrier plate at the density of $1x10^6$/well and macrophages were cultured into the insert at the density of $0.5x10^6$/well. After 3-day co-culture, T cells were isolated for analyses.

RNA-seq data analysis

The raw data of sequencing were processed by trim_galore v0.6.5, and then reads were mapped using STAR v2.6.1d with mm10 reference genome. R and edgeR package were used to calculate differential expression. P-values were analyzed for multiple testing by the false discovery rate (FDR) correction of Benjamini and Hochberg. R and package clusterProfiler were selected to perform gene ontology biological process enrichment analysis.

Statistics

Statistical analysis was conducted on normally distributed data sets. Representative data are presented as the mean ± SD. Sample size (n) was provided in figure legends. GAPDH was used for the normalization of qPCR data and β-actin was used for western blot data normalization. The student's two-tailed *t*-test was used for the statistical significance of two group comparisons. A one way or two-way ANOVA was performed for the statistical significance of three or more group comparisons. The log-rank test was conducted for the statistical significance of animal survival rates. $P < 0.05$ was considered to be statistically significant. SPSS 17.0 software was used to perform statistical analyses.

Reference

[1] L. Q. Qiu, D. J. Stumpo, P. J. Blackshear, *Journal of immunology* **2012**, *188* (10), 5150, https://doi.org/10.4049/jimmunol.1103700.

[2] J. Yao, D. Wu, C. Zhang, T. Yan, Y. Zhao, H. Shen, K. Xue, X. Huang, Z. Wang, Y. Qiu, *Nature immunology* **2021**, *22* (10), 1268, https://doi.org/10.1038/s41590-021-01023-y.

[3] S. Wirtz, V. Popp, M. Kindermann, K. Gerlach, B. Weigmann, S. Fichtner-Feigl, M. F. Neurath, *Nature protocols* **2017**, *12* (7), 1295, https://doi.org/10.1038/nprot.2017.044.

[4]D. J. Berg, N. Davidson, R. Kuhn, W. Muller, S. Menon, G. Holland, L. Thompson-Snipes, M. W. Leach, D. Rennick, *The Journal of clinical investigation* **1996**, *98* (4), 1010, https://doi.org/10.1172/JCI118861.

[5] T. Nguyen, J. Du, Y. C. Li, *STAR protocols* **2021**, *2* (4), 101004, https://doi.org/10.1016/j.xpro.2021.101004.

[6] F. L. Szeto, C. A. Reardon, D. Yoon, Y. Wang, K. E. Wong, Y. Chen, J. Kong, S. Q. Liu, R. Thadhani, G. S. Getz, Y. C. Li, *Molecular endocrinology* **2012**, *26* (7), 1091, https://doi.org/10.1210/me.2011-1329.

[7] J. Goc, M. Lv, N. J. Bessman, A. L. Flamar, S. Sahota, H. Suzuki, F. Teng, G. G. Putzel, J. R. I. L. C. Bank, G. Eberl, D. R. Withers, J. C. Arthur, M. A. Shah, G. F. Sonnenberg, *Cell* **2021**, *184* (19), 5015, https://doi.org/10.1016/j.cell.2021.07.029.

[8]J. Du, W. Liao, W. Liu, D. K. Deb, L. He, P. J. Hsu, T. Nguyen, L. Zhang, M. Bissonnette, C. He, Y. C. Li, *Developmental cell* **2020**, *55* (6), 737, https://doi.org/10.1016/j.devcel.2020.10.023.

[9] K. Atarashi, T. Tanoue, T. Shima, A. Imaoka, T. Kuwahara, Y. Momose, G. Cheng, S. Yamasaki, T. Saito, Y. Ohba, T. Taniguchi, K. Takeda, S. Hori, Ivanov, II, Y. Umesaki, K. Itoh, K. Honda, *Science* **2011**, *331* (6015), 337, https://doi.org/10.1126/science.1198469.

[10]Y. Zheng, P. A. Valdez, D. M. Danilenko, Y. Hu, S. M. Sa, Q. Gong, A. R. Abbas, Z. Modrusan, N. Ghilardi, F. J. de Sauvage, W. Ouyang, *Nat Med* **2008**, *14* (3), 282,

https://doi.org/10.1038/nm1720.
[11]Y. Shi, T. Liu, L. He, U. Dougherty, L. Chen, S. Adhikari, L. Alpert, G. Zhou, W. Liu, J. Wang, D. K. Deb, J. Hart, S. Q. Liu, J. Kwon, J. Pekow, D. T. Rubin, Q. Zhao, M. Bissonnette, Y. C. Li, *Scientific reports* **2016**, *6*, 27552, https://doi.org/10.1038/srep27552.
[12]H. Huang, H. Weng, W. Sun, X. Qin, H. Shi, H. Wu, B. S. Zhao, A. Mesquita, C. Liu, C. L. Yuan, Y. C. Hu, S. Huttelmaier, J. R. Skibbe, R. Su, X. Deng, L. Dong, M. Sun, C. Li, S. Nachtergaele, Y. Wang, C. Hu, K. Ferchen, K. D. Greis, X. Jiang, M. Wei, L. Qu, J. L. Guan, C. He, J. Yang, J. Chen, *Nature cell biology* **2018**, *20* (3), 285, https://doi.org/10.1038/s41556-018-0045-z.
[13]J. Wei, X. Yu, L. Yang, X. Liu, B. Gao, B. Huang, X. Dou, J. Liu, Z. Zou, X. L. Cui, L. S. Zhang, X. Zhao, Q. Liu, P. C. He, C. Sepich-Poore, N. Zhong, W. Liu, Y. Li, X. Kou, Y. Zhao, Y. Wu, X. Cheng, C. Chen, Y. An, X. Dong, H. Wang, Q. Shu, Z. Hao, T. Duan, Y. Y. He, X. Li, S. Gao, Y. Gao, C. He, *Science* **2022**, *376* (6596), 968, https://doi.org/10.1126/science.abe9582.
[14]J. Du, Y. Chen, Y. Shi, T. Liu, Y. Cao, Y. Tang, X. Ge, H. Nie, C. Zheng, Y. C. Li, *Inflammatory bowel diseases* **2015**, *21* (11), 2495, https://doi.org/10.1097/MIB.0000000000000526.
